# Supplementary material for: Health care during electricity failure: The hidden costs
Source: PLoS One. 2020 Nov 4;15(11):e0235760. doi: 10.1371/journal.pone.0235760 (PMC7641375; doi:10.1371/journal.pone.0235760)
Supplement: S1 File — (PDF) [file pone.0235760.s001.pdf]

Health Care during Electricity Failure: The Hidden Costs

Abigail Mechtenberg[1,2,*], Brady McLaughlin[1,2], Michael DiGaetano[1,2], Abigail Awodele[1,2], Leslie Omeeboh[1,2], Emmanuel Etwalu[3], Lydia Nanjula[4], Moses Musaazi[3†], Mark Shrime[5]

**1** Center for Sustainable Energy, University of Notre Dame, South Bend, Indiana, United States
**2** College of Science, Department of Physics and Preprofessional Studies, University of Notre Dame, South Bend, Indiana, United States
**3** College of Engineering, Design, Art, and Technology (CEDAT), Makerere University, Kampala, Uganda
**4** Mulago Hospital, Kampala, Uganda
**5** Harvard Medical School, Harvard University, Boston, United States

¤Current Address: Dr. Mechtenberg at Physics Department, 208 Jordan Hall of Science, University of Notre Dame, Notre Dame, Indiana, 46556, United States or * amechten@nd.edu.
†Deceased
¶Membership list can be found in the Acknowledgments section.

# Supporting information

Due to the undisputed fact that LMICs have little electricity reliability, the medical community should have a procedure in place to systematically address this concern in terms of a cost-benefit analysis or risk analysis, but nothing substantial exists [?]. This Energy Healthcare System (EHS) model allows medical professionals (1) to estimate the percentage of procedures in each of the four impact groups based on experience at the hospital and calculate the VSL/E based on patient deaths modeled to argue for more backup energy systems to increase hybridization and (2) to argue for an energy storage monitoring system where they are performing medical procedures in rooms with check lists of how much energy is required to perform specific medical procedures as well as check lists for procedures to implement if and when there is a failure. The first option is a global medical recommendation that hospitals consider Levelized Cost of Electricity as well as Value of a Statistical Life Lost due to the electricity shortage. The second option is a local medical recommendation that hospitals implement two types of check lists.

## 0.1 Data on Four Energy Healthcare System (ESH) Types and Methodology to Calculate Risks, Failure Events, and Costs

This section describes the two methodologies created to connect electricity failure and additional patient risks due to these failures, in greater detail. The authors obtained energy failure events for a year (8760 hours) for four health care facilities from Homer Energy optimal models and/or measured data [?] [?] [?] [?] [?] [?] [?]. These show differences between countries and between energy system types. Three of these were simulations, originally published in research journals [?] [?] [?] [?]. The fourth is based on real measurements of voltage and current where every 15 minutes a data point averaging the measurements was sent to a server via satellite. They all have unique research papers

proposing various solutions, but all of the energy solutions presented do not eliminate electricity failures [?] [?] [?] [?].

From the analysis of yearly energy events for four health care facilities, the authors created four patient impact models: no, low, medium, and high additional patient risk due to electricity failure. Below is a snapshot of how the year events were analyzed, and the focus on said analysis.

**S1 Fig. Electricity Failure Events (red) for Iraq Rural Health Care Facility published in Solar Energy, 2010 [?].** Hours of the day on the y-axis and days of the year on the x-axis with color showing state of electricity system: on (blue) or off (red).

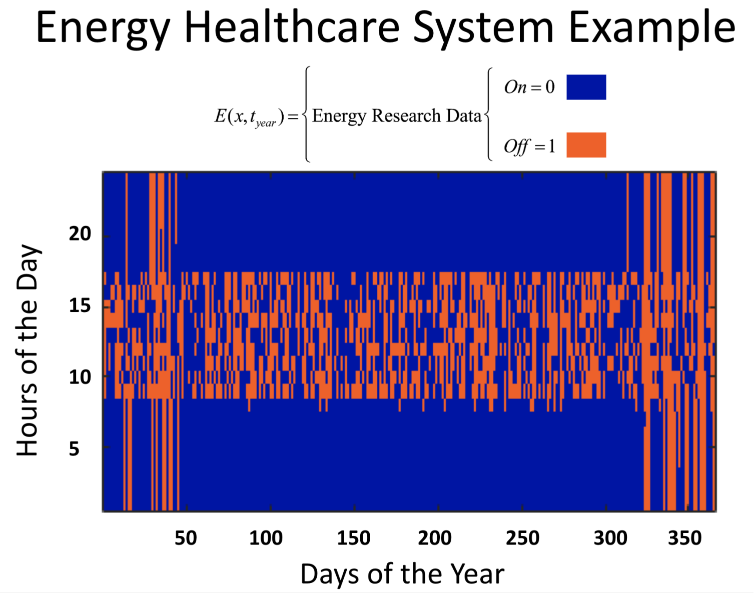


## 0.2 Methodology and Data for Additional Patient Risk for Four EHS Types

In Figure S1, 8760 electricity data points, modeled and optimally chosen for a solar-powered hospital facility in Iraq [?] are plotted in a binary fashion to signify the state of the energy healthcare system's state: either electricity is on or off. Each data point reflects each hour of the day for a year. The points are categorized by whether electricity was on (energy value is zero and blue) or off (energy value is one and red). These values are used for the calculations of additional risk later on in the paper.

As can be seen, this solar-powered health care facility faces single long-duration and multiple short-duration power failures and the authors define this as EHS-Type 1. These failures are longer in duration during the "wet season" due to more extensive cloud coverage, but will occur whenever the power load depletes the battery, even during the "dry season" when the solar radiance is at peak levels, the system fails randomly. The categorization of these seasons is evident, as the wet-season comprises the first and last few months of the year (before day 50 and after day 300 in the year), whereas the dry season comprises the rest of the year. It is imperative to understand that during the time of the day when the power load is greatest, the batteries are often depleted in one hour and then recharged the next hour. This means that medical professionals experience random electricity failures throughout the day, reflected qualitatively in previous research literature. The next section describes the details of each EHS-Type in detail.

### 0.2.1 Countries and Details on Energy Healthcare Systems Types

Acakpovi and Hagen [?] [?] published an article, in International Journal of Computer Applications, comparing various optimization algorithms for cost optimizing electricity energy systems, using Homer Energy. They make an interesting policy comment about hydroelectricity providing for base power, potentially from the government centralized grid, while leaving isolated mini grids for solar and wind hybrid installations to meet peak power. The specifically talk about 50% capacity shortage means that the government covers 50% and the local institutions will only have to cover the other 50%. Although they do not directly mention health care facilities, this model highlights the extreme differences between energy and time capacity shortages that can arise which support qualitative reports from health care facilities that "electricity fails about half the time."

This inspired the authors analysis of over 120 academic papers that used the Homer Energy mini grids optimization software program to design an optimal energy system. Evaluation focused on health care design, geographical location, socioeconomic status, and reproducibility of results. Of these academic papers, four published results are highlighted to underscore capacity shortage, energy system type, and LCOE tradeoffs in terms of additional patient risk not considered. In these four papers, the energy system in Iraq-SolarPV [?], Bangladesh-SolarPV+Wind [?] [?], Ghana-Hydroelectric [?], and Uganda-Grid+Diesel generator [?] were optimally designed with greater than 5% capacity shortage assumed. They were considered scientifically valid and peer-reviewed publication applicable based on LMICs, within the given the constraints for methodology, and/or argued for local health care facilities to design a backup system to meet capacity shortage.

### 0.2.2 Iraq and Bangladesh Optimal Energy Healthcare Systems for Rural Health Care Facilities

Al-Karaghouli and Kazmerski [?] published, in Solar Energy, a state-of-the-art rural health care energy system in Iraq that included solar panels, batteries, and a converter. It is noteworthy that many government aid programs have installed these types of energy healthcare systems throughout LMIC's and what is defined as EHS-Type 1. As such, this paper highlights a telling health care energy system phenomenon, unfolding a pseudo case study design. Nandi and Ghosh [?] published, in Energy, another rural health care energy system for southeastern Bangladesh with wind turbines, solar cells, batteries, and converter. In both cases, the optimal energy system chosen had a capacity shortage. There was no discussion of options for a back-up energy system, that could be locally maintained and is on-demand for critical, health care power loads, in case of electricity failure.

**S2 Fig. Comparison of Energy and Time Capacity Shortages for Iraq and Bangladesh Rural Health Facilities.** The optimal systems chosen in the research articles were solar only for Iraq, and wind and solar for Bangladesh.

## Electricity & Healthcare Systems Summary (EHS-Type 1 and EHS-Type 2)

| | Iraq - *Solar Energy,* 2010 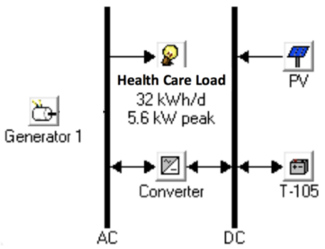  | Bangladesh - *Energy*, 2010 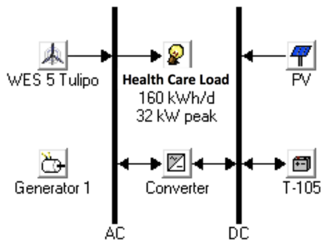  |
|---|---|---|
| Cost of Electricity ($/kWh) | $0.26/kWh | $0.51/kWh |
| Energy Capacity Shortage (%) $\frac{E_{not\ served}}{E_{total}}$ | 18 % | 10 % |
| Time Capacity Shortage (%) $\frac{t_{not\ served}}{t_{total}}$ | 25 % | 6.6 % |
| Failures (hrs/year) | 2160 hours | 574 hours |
| Back-up System | Install Solar-Battery-Converter no backup diesel generator chosen | Install Solar+Wind-Battery-Converter no backup diesel generator chosen |

Figure S2 illustrates that in both of the EHS-Types, there still exists capacity shortage. In Iraq the localized cost of electricity (LCOE) was $0.26/kWh with an energy capacity shortage of 18% and a time failure of 2160 hrs/year (or 25% of the time). A common question that arose during this analysis was this: "why not just have a back-up diesel generator?" The authors of this paper argue that it is impractical to bring a diesel generator due to the added difficulty in transporting the fuel to the remote health care center. Although there are other options, however, these authors did not discuss the other options or what should happen when their is an electricity failure (as discuss in Section 5 of this paper). In Bangladesh the LCOE is $0.51/kWh with an energy capacity shortage of 10% and a time failure of 574 hrs/year (6.6% of the time). The authors did not include a diesel generator as an option because of the extra expense which would increase energy costs. Although there are otherno diesel generator options, these authors did not discuss the other options or what should happen when their is an electricity failure (again as discuss in Section 5 of this paper). In both cases, no discussion was given in terms of the cost of patient lives lost due to an electricity failure. One methodological note is that the community in Bangladesh installing this energy system would be powering a healthcare facility, although not directly discussed by authors.

### 0.2.3 Ghana and Uganda Optimal Energy Healthcare Systems for Regional Health Care Facilities

Acakpovi and Hagen [?] [?] published an article in International Journal of Computer Applications comparing various optimization algorithms for cost optimizing electricity energy systems showing that Homer Energy positive robust, reliable attributes while also making an interesting policy comment about how hydroelectricity can provide for base power potentially from the government centralized grid while leaving isolated minigrids for solar and wind hybridizations installations to meet peak power as mentioned earlier and reiterated here. Although they do not talk directly about health care facilities, reproducing this model highlights the extreme differences between energy and time

capacity shortage can arise and included due to qualitative reports from health care facilities that the “electricity fails about half the time”. Mechtenberg et. al [?] published in Sustainable Systems and Technology (ISSST-IEEE) the measured data for a regional hospital with grid access and a back-up diesel generator to discuss the potential backup devices that could be considered during electricity failures as well as the potential for human rights violations associated with avoidable unmet loads defined as avoidable and unnecessary suffering. Considering the recent legal movement against an electric utility to document human rights violations, this is vitally important for discussion and consideration (see Section 4 and the discussion about UMEME).

**S3 Fig. Comparison of Energy and Time Capacity Shortages for Ghana and Uganda Urban Energy Systems.** The optimal systems chosen in the research articles were hydroelectric for Ghana with and grid and diesel generator for back-up in Uganda with a discussion about the reasons for a small acceptable capacity shortage.

## Electricity & Healthcare Systems Summary (EHS-Type 3 and EHS-Type 4)

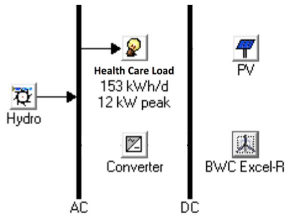


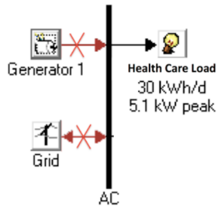


| | Ghana - *International Journal of Computer Applications,* 2015 | Uganda - *ISSST-IEEE*, 2012 |
|---|---|---|
| Cost of Electricity ($/kWh) | $0.12/kWh | Grid: $0.25/kWh - Diesel: $0.75-11/kWh |
| Energy Capacity Shortage (%) $\frac{E_{not\ served}}{E_{total}}$ | 11 % | 4.1 % |
| Time Capacity Shortage (%) $\frac{t_{not\ served}}{t_{total}}$ | 49 % | 4.0 % |
| Failures (hrs/year) | 4289 hours | 355 hours |
| Back-up System | Government Grid: Hydro base power, Facilities: Provide their own peak power | Year of Data on Voltage and Current Grid+Diesel Generator |

Like in Figure S2, Figure S3 also shows capacity shortages in both EHS-Types. In the Ghanaian hydroelectric energy system for base power, the LCOE was $0.12/kWh with an energy capacity shortage of 49% and a time failure of 4289 hrs/year (or 49% of the time). The authors of this paper seem to argue that isolated mini-grid systems (including health care facilities) should rely on the centralized electricity system for base power load from a hydroelectric only system. There is also another implication that they design their own site-based solar and wind backup isolated mini-grid energy systems. Although there is a belief, as discussed by the authors of the Ghanaian energy paper, that when water is available, there will be affordable electricity because hydro is extremely cost effective, certain constraints still exists for governments of LMICs' in designing hydroelectricity systems to provide for base power, leaving peak power as unattainable (or due to growth in peak power). In Uganda the LCOE is $0.25/kWh for grid with costs for diesel generator above $0.75/kWh and as high as $11/kWh when idling the diesel generator [?]. This EHS-Type is unique in that it is actual, measured

data of voltage and current for a year (with multiple measurements taking in a 15 minute time space and the four 15 minute measurements averaged for the hour time space) with an energy capacity shortage of 4.1% and a time failure of 355 hrs/year (4.0% of the time). In both cases, no discussion was given in terms of the cost of patient lives if electricity fails during health care procedures within the community or in a specific regional hospital.

## 0.3 Energy Healthcare System Type Data and Matrix

Many grid and electrical energy systems in LMICs have been designed with the acceptance or understanding of having between a five to twenty percent capacity shortage (with many locations having higher capacity shortages), and with the distribution being heavily skewed toward the latter. By compiling energy data taken over the course of a year, the duration of electrical failures can be quantified and plotted across the year.

$$\mathrm{E_{Type}}(\mathrm{t_{day}}, \mathrm{t_{hour}}) = \begin{cases} 0 : Electricity - On \\ 1 : Electricity - Off \end{cases} \tag{1}$$

Equation 1 shows the only two options for the electricity state matrix element data for a given EHS-Type, $E_{Type}$, in terms of day of the year, $t_{day}$, and hour of the day, $t_{hour}$. The electricity is either on, in which case there is no additional risk to patients, or off, when the risk function will be calculated. These capacity shortages are typically presented as a single number, in the research literature, and not as a yearly matrix plot (Hours during the Day versus Days of the Year). To illustrate this point, a common 20% capacity shortage would be presented as 80% electrical power. Furthermore, previous researchers have noted that decreases in capital costs of healthcare energy systems can be 50% when a 20% capacity shortage is accepted without another cost-benefit analysis. Consequently, the medical consequences of these 20% electricity failures in a health facility will not be calculated because it is complicated. The complications involve dependence on the type of medical procedure, time of failure, duration of failure, as well as frequency of failure events. Therefore, it is imperative to consider four broad and distinct EHS-Types.

The research literature, presented above, portrays the unreliability of electricity for a wide variety of health care facilities even though with grid, back-up diesel generator and even facilities designated as having a state-of-the-art solar energy system. Regardless of the energy system and location, these models clearly illustrate the reliability issues throughout the year. Furthermore, they package the qualitative reporting of medical professionals into a quantifiable data aggregate. Figure S4 graphs the time of failures for these four energy healthcare system types based on published optimization models and/or actual measured data, as discussed above.

**S4 Fig. Comparison of electricity failure pattern results for four EHS-Types.** They represent four LMIC regions (Iraq, Bangladesh, Ghana, and Uganda) as well as four common energy system types (solar only, solar and wind hybridization, hydroelectric generator for base power, and utility grid with backup diesel generator).

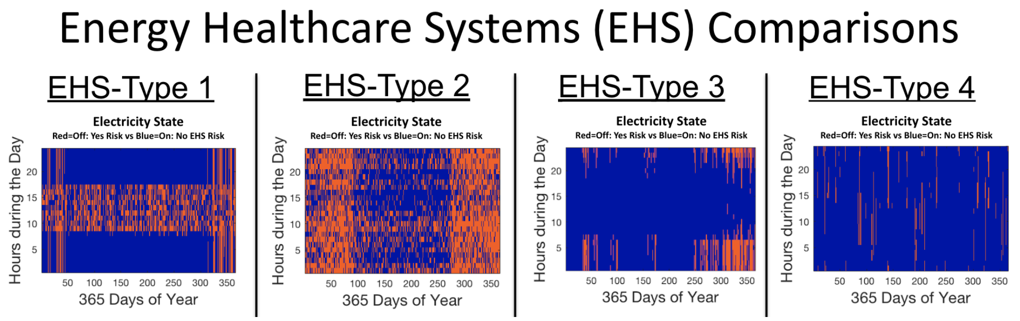


These failure events do not inform the medical professional about the risks to patients, overall effects to specific medical procedures and health care, nor how much more the medical facility should spend to decrease the failure events. In the next subsection, the authors of this paper created a model to calculate the additional risks to patient based on time of electricity failure and duration of failure as well as modeled patient deaths based on the size of health care facility and whether a medical procedure had been initiated before the failure event. This allows for facilities to calculate the number of patients deaths and costs, based on this model, to decide if additional backup energy systems should be considered.

## 0.4 Patient Additional Risk due to Failure

Medical procedures that depend on electricity mean that patients needing those medical procedures have additional risks solely due to electricity failure. The authors have created a quantifiable model of these additional patient risks based on time of electricity failure and type of medical procedure. The equation for additional patient risk is as follows:

$$\mathrm{r_{ij}}(\mathrm{t_d}) = \frac{1}{1 + exp(-k_j(t_d - c_i)} \tag{2}$$

$$\begin{cases} i : 1 \text{ to } 3 \text{ for low, medium, and high impact medical procedure} \\ j : 1 \text{ to } 3 \text{ for minimum, mean, and maximum for uncertainty in risk} \\ c_i : \text{ time at which risk is } 50\% \\ k_j : \text{ slope of the risk function for the time in which risk is } 50\% \\ t_d : \text{ duration of electricity failure (in hours)} \end{cases}$$

Equation 2 shows the nine logistic functions for the additional risk to patient model, $r_{ij}(t_d)$, in terms three medical procedure impact levels, $i$, in terms three statistical metrics, $j$, and duration of the electricity failure, $t_d$. This additional risk function is based on $c_i$, the time at which the patient has a fifty percent chance of survival (0.5 additional risk), $k_j$ the slope of the function for the time in which the patient's chance of survival is fifty percent, and is a commonly used two parameter risk function that only depends on duration time of failure. This function goes from zero, no additional risk to patient, and approaches 1, absolutely certainty the patient dies, but the function never equals 1. Basically, the additional risk grows the longer the duration of the electricity failure, but there is never the opportunity to know the patient is dead so it approaches a probability where it is highly likely the patient dies.

There are a total of nine curves which are easier to understand graphically, as seen in Figure S5 as three sets of impacts each have three uncertainty level curves that depend on duration of electricity failure, $t_d$, and estimations of the two parameters, $c$ and $k$.

**S5 Fig. Additional patient risk functions parameters used in equation 2 to generate nine additional patient risk curves that depend on medical procedures that have varying impacts due to electricity failure (low, med, high) and that depend on an uncertainty range for the probability (min, mean, and max).** Y-axis presents the additional risk to patient as a result of starting a medical procedure which required an electrically powered medical device. The duration of the electricity failure is on the x-axis. Medical procedures can be placed into three categories of impact regions (high impact - red, medium impact - green, and low impact - blue).

| $k_{High}$, $c_i$ | $k_{Med}$, $c_i$ | $k_{Low}$, $c_i$ |
|---|---|---|
| $(k_1,c_1)=(1,0.1)_{min}$ | $(k_2,c_1)=(1.5,2)_{min}$ | $(k_3,c_1)=(2,4)_{min}$ |
| $(k_1,c_2)=(1,3.0)_{mean}$ | $(k_2,c_2)=(1.5,5)_{mean}$ | $(k_3,c_2)=(2,7)_{mean}$ |
| $(k_1,c_3)=(1,6.0)_{max}$ | $(k_2,c_3)=(1.5,8)_{max}$ | $(k_3,c_3)=(2,10)_{max}$ |

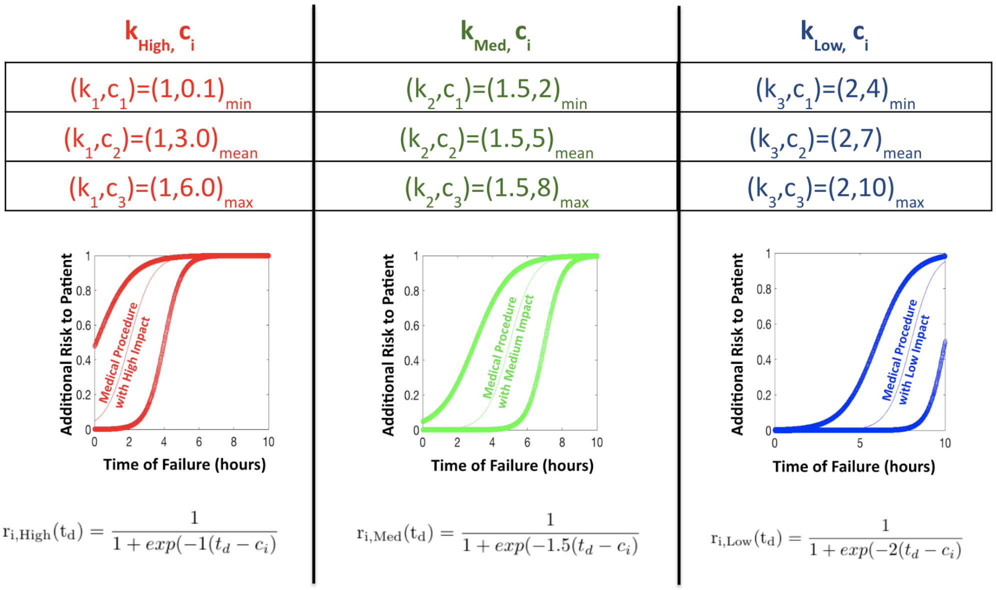


$$r_{i,High}(t_d) = \frac{1}{1+exp(-1(t_d - c_i)}$$

$$r_{i,Med}(t_d) = \frac{1}{1+exp(-1.5(t_d - c_i)}$$

$$r_{i,Low}(t_d) = \frac{1}{1+exp(-2(t_d - c_i)}$$

**Note:** six more probabilistic two parameter functions modeled as well. Email for more details - risk model is adaptable, flexible, and verifiable.

The three groups of curves in Figure S5 represent the consequences of starting a medical procedure requiring electricity after which an electrical failure occurs. The patient experiences additional risks, not previously been quantified by medical research (mildly impacted in blue, moderately impacted in green, or highly impacted in red). It is interesting to note that medical procedures that have high impact due to electricity failure typically have higher energy use than low impact, although this is not always the case.

## 0.5 EHS-Type Matrix and Risk Function into One Methodology

The authors combined both methodologies: additional patient risk model and the energy failure simulation events and/or measured data. Together, they enabled the calculation of risk to patients due to electricity failure assuming a medical procedure had been started that requires electricity (deaths per 1,000 patients), and the cost in the Value of Statistical Lives lost divided by energy shortage ($/kWh).

### 0.5.1 Calculating Risk Matrix by Combining the Two Methodologies

There are many types of medical procedures that require electricity powered devices. Based on interviews from surgeons, and the assumption of the need to increase the number of global surgical wards internationally, the medical procedure first considered for this investigation was surgery. To calculate additional risks, the time of medical procedure and duration of electricity outage need to be examined. Furthermore, there needs to be an understanding of the logic of recording additional risk to patients during

the year, based on the time and duration of the electricity failure, if and/or when it happens.

**S6a Fig. Four EHS-Type Electricity Failure Matrices for every hour of the day and every day of the year**

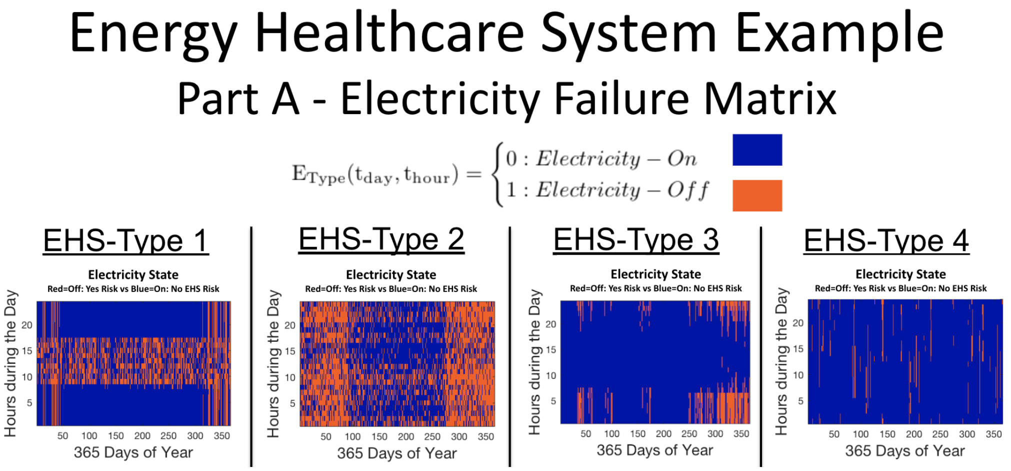


**S6b Fig. Nine Risk Functions based on Medical Procedures Impacts due to Electricity Failures and Confidence in Impact Groups**

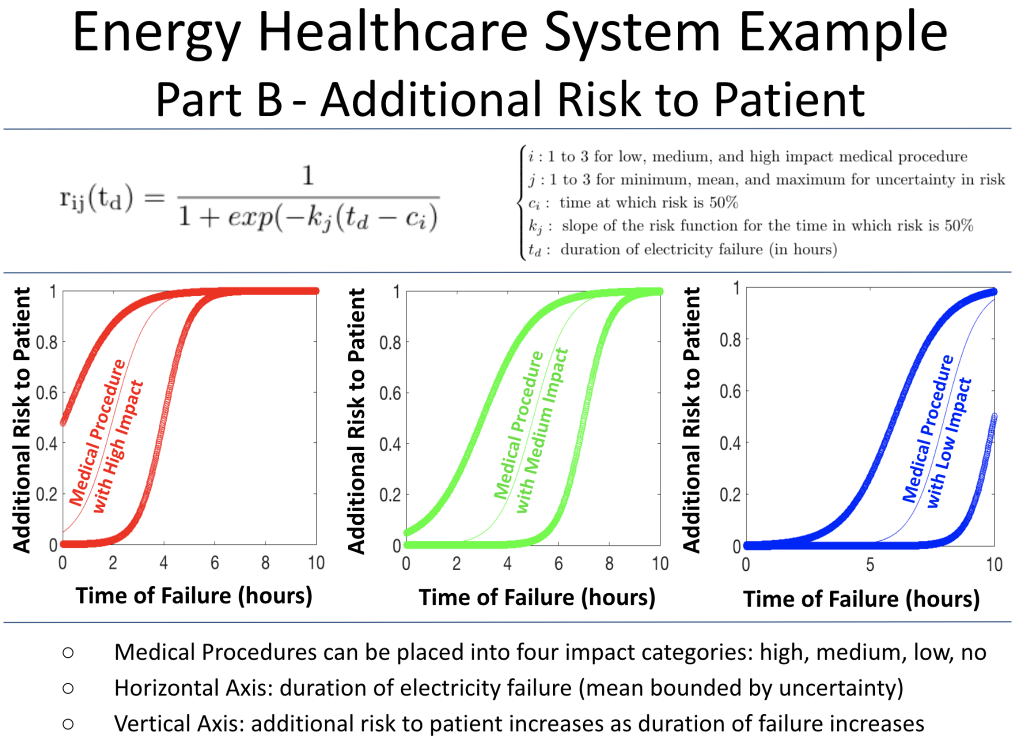


In combining the two methodologies into one methodology, there were two steps. First energy healthcare system type, the electricity state and risk function is multiplied together to calculate the risk for a given medical procedure in a particular impact group is calculated for the entire year.

$$R(E_{Type}, r_{ij}) = E_{Type}(t_{day}, t_{hour}) r_{ij}(t_{duration}) \quad (3)$$

As time moves forward, the duration of failure is recorded as outlined in this flow chart of logic.

**S7 Fig. Logic to Calculate the Risk based on the Duration of the Electricity Failure**

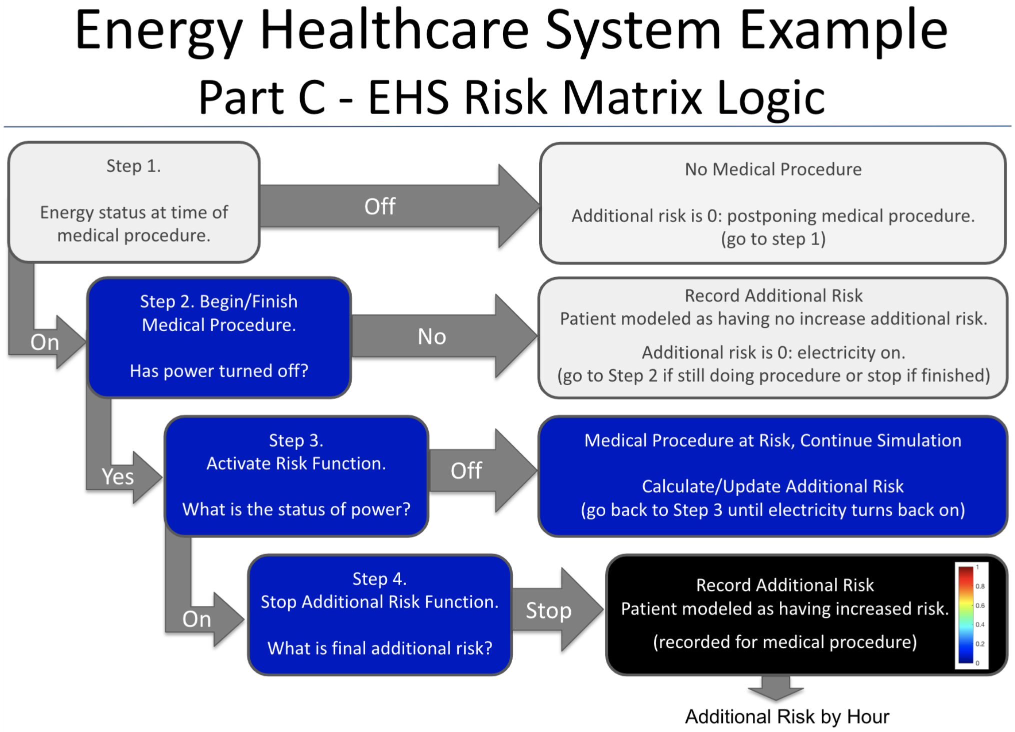


A key part of this methodology, is to implement the basic knowledge that if electricity is off, then a new medical procedure is postponed. This means that all no new medical procedures would have been started and therefore these entries in the matrix are deleted before calculating the total risk. Therefore, Figure S7 shows that additional risk is only calculated if the electricity fails and a medical procedure was started. If a medical procedure is scheduled to start and the electricity is off, then it is assumed that the procedure is postponed. This happens often and is addressed in this analysis by deleting these hours out of the matrix.

### 0.5.2 Example Risk Matrix when Combining the Two Methodologies

For a generic energy healthcare system type where electricity fails from 2pm to 8pm, Figure S8 shows the energy system for the year and the 3x3 matrix for the nine additional patient risks.

**S8a Fig. The three medical impact level types (high, medium and low impact) have a mean calculation as well as minimum and maximum to bound the confidence interval to estimate uncertainty in additional risk which results in a 3x3 matrix of risks during the year due to electricity failure and du-**

**ration of failure.**

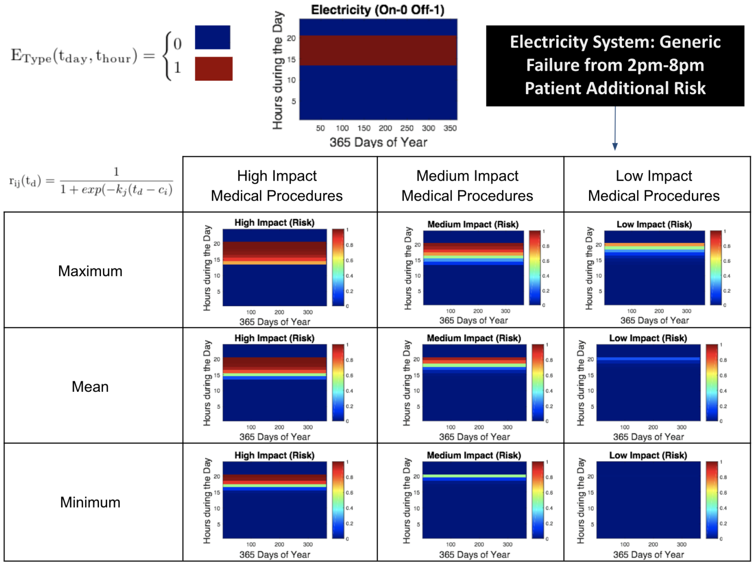


**S8b Fig. The final Risk Matrix deletes all the medical procedures which would have been postponed due to previous hour(s) of electricity failure(s). Also, this represents a EHS-Type 0 called the Generic Electricity Failure pattern from power load shedding where the government and/or utility company posts the times when specific cities will or will not have electricity during the day. This EHS-Type was not considered in the initial risk chart groups because medical professionals postpone medical procedures when there is knowledge of an upcoming electricity failure.**

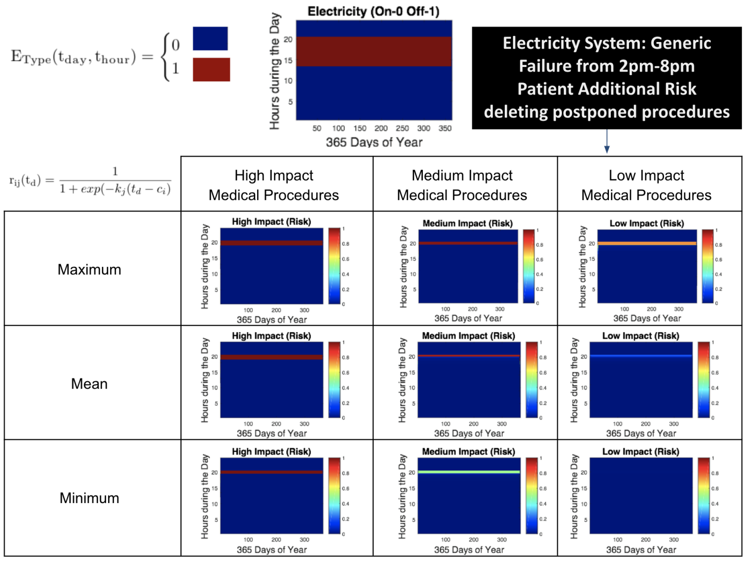


Figures S8a and S8b show the differences between impact groups and additional risk to patient based on duration of electricity failure for this hypothetical generic EHS type facility. For example, when the medical procedure is highly dependent on electricity, the additional risk to patient moves from 0.10 to 0.25 when the duration of electricity is 1 to 2 hours, with a confidence interval between 0 and 0.65. However, the low impact medical procedures would be close to zero for 1 to 2 hours duration with a confidence interval

between 0 and 0.01. This additional patient risk model calculates uncertainties for high, medium and low impact medical procedure. Future work will be based on collected data such that specific medical procedures are placed into appropriate impact groups based on the results of patient outcomes when electricity fails.

So the overall risk for the year is based on the average over the year of the risk each hour. For the mean of the medical procedures that have high impacts due to electricity failure, there are only 52 hours in the year (one hour a day) where the risk is around 0.99 and the rest of the 8,708 hours in the year there was recorded no risk. This no risk is either due to other procedures being postponed or because the electricity was on. So, the mean high impact risk result is $(8,708 * 0.00 + 52 * 0.99)/8760$ or 5.9 deaths per 1,000 patients. Whereas the mean low impact group for this long 6 hour failure every day of the year, would have a risk result of $(8,708 * 0.00 + 52 * 0.20)/8760$ or 1.2 deaths per 1,000 patients for the year. Overall, this is a very conservative risk model by deleting all medical procedures that would have been postponed, but the more important aspect to the model is its ability to be flexible, adaptable, and verifiable.

## 0.6 Calculating Hidden Costs

A cost-benefit analysis of an energy healthcare system requires an economic value of a health care facilities' energy system reliability and this has not been systematically accomplished in previous literature. The two parts of this methodology, have been extensively, and individually, discussed in detail above. However, a combined cost-benefit analysis requires understanding the costs associated with these failures and risks. As discussed in Mechtenberg et [?] there was already a backup diesel generator system included in the Uganda Regional Hospital energy system, but there were still time and energy capacity shortages. This means that there is no way to meet critical loads in theatre (surgical ward) when these failures occurred, leading to deaths that could be avoided. How does a medical facility know how to execute a cost-benefit calculation of whether or not to consider an additional backup system for critical loads? Currently, there is no quantifiable methodology and where there two or more ethical scenarios to consider without any data or quantifiable models to base the medical decision on only anecdotal experience from experts evaluate alternatives, assuming there is time and adequate understanding of risks (chance of death and duration of electricity failure)9. Furthermore, in the energy system design literature, people tend to calculate the Levelized Cost of Electricity over the lifetime of the energy system in terms of total cost and total energy consumed (LCOE: \$/kWh). There needed to be a clear way to compare an energy system cost due to not providing electricity. This section describes the authors' metric to compare to LCOE to understand if an additional backup system should be installed at a health care facility.

### 0.6.1 Defining Cost of VSL/E due to Capacity Shortage

Considering the energy system designs and additional risk to patients calculated over the year in the previous section, the authors documented the Value of a Statistical Life (VSL) in these four countries and a new term the authors call the Value of a Statistical Life Lost per Energy Capacity Shortage (VSL/E). Traditional VSL is amount of money that people are willing to pay for a marginal decrease in their own mortality due to the probability of a patient dying when electricity fails. Costs of electricity failures are typically framed as an economic externality when designing an energy system for health care centers in LMICs. It is vital that this economic externality be internalized when designing an energy system for health care as shown by these four VSL/E calculations.

The Value of a Statistical Life is a well documented idea in economics, as well as in the recent discussions of Global Surgery, where it is defined by measuring out how

much people are willing to pay for small decreases in the risk of death and extrapolating out to an entire life. The research suggests using different elasticities for the VSL of LMICs, and recommends using elasticities (n) of 1.0, 1.5, and 2.0, which were used to calculate three different VSLs for each of the four nations examined here, giving a total of 12 different VSLs. The well-established economics equation follows:

$$(\mathrm{VSL_n})_{\text{target country}} = (VSL)_{\text{known country-US}} \left[ \frac{(\mathrm{GDP/Capita})_{\text{target country}}}{(\mathrm{GDP/Capita})_{\text{known country-US}}} \right]^n \tag{4}$$

Given equation 4, the following table presents our VSL results for Iraq, Bangladesh, Ghana, and Uganda in Table S1.

**S1 Table. Values of a Statistical Life for the countries under study based on different elasticities.**

| | $\mathrm{VSL_1}$ | $\mathrm{VSL_{1.5}}$ | $\mathrm{VSL_2}$ |
|---|---|---|---|
| Iraq (2006$) | $339,649.60 | $711,956.44 | $1,492,362.30 |
| Bangladesh (2006$) | $23,021.00 | $94,227.00 | $365,684.00 |
| Ghana (2006$) | $31,055.23 | $118,362.60 | $451,264.97 |
| Uganda (2006$) | $2,430.77 | $17,404.29 | $122,899.56 |

Applying this VSL value directly to electricity, if a hospital installs a back-up electricity system in the amount of money equal to the VSL of that country and it results in saving one life, then it is considered an economically sound decision when the electrical capacity shortage is 1 kWh because these elasticities are much greater than $1/kWh (a high LCOE). Likewise, when one or more lives can be saved, the added benefit dramatically increases. However, if there is a lot of electricity needed say (1,000,000,000 kWh) for one life to not statistical lose a life, then it would definitely not be cost beneficial to spend the money to add that backup energy system.

The value of a statistical life lost due to electricity failure and the amount of electricity shortage are two variables considered. These two variables create the cost due to electricity failure for a new metric called VSL/E, which is the cost of electricity when taking into account the value of a statistical life ($) lost because of a capacity shortage, measured in electricity energy units of kilowatt hours (kWh).

$$(\mathrm{VSL_n})/\mathrm{E_{shortage}} = \frac{\mathrm{VSL_n} * \text{Lives Lost}}{\text{Energy Capacity Shortage(kWh)}} \tag{5}$$

Using equation 5 with at least one patient death due to electricity failure during a medical procedure requiring electricity and the capacity shortage from Homer Energy model, Table S6 presents the VSL/E in terms of $/kWh. Since three VSLs were calculated for four countries, there were 12 different VSL/E for each scenario of patient deaths based on 3 elasticities.

**S2 Table. The VSL/E values associated with 1 death per year associated with the EHS-Type's electricity shortages in each country compared to LCOEs in research papers used given the uncertainties in elasticities.**

| VSL/E ($/kWh) with 1 patient death and 3 elasticity estimations | | | |
|---|---|---|---|
| **Country (COE: $/kWh)** | **$VSL_1/E$** | **$VSL_{1.5}/E$** | **$VSL_2/E$** |
| Iraq ($0.26/kWh) | $267.88/kWh | $561.52/kWh | $1,177.03/kWh |
| Bangladesh ($0.51/kWh) | $5.16/kWh | $21.14/kWh | $86.52/kWh |
| Ghana ($0.12/kWh) | $6.92/kWh | $26.36/kWh | $100.50/kWh |
| Uganda ($0.25/kWh) | $5.37/kWh | $38.42/kWh | $271.30/kWh |

Given the results in Table S2 for one patient death, it is clear that there is a disconnect between LCOE and reality of additional risks to patients when electricity failures. No health care facility should use LCOE solely as a metric for designing a cost efficient energy system for a hospital. Even if all electricity is generated in one year, when a LMIC health care facility grows, the capacity shortage grows and immediately backup energy systems should be in place and designed based on power load prioritizations to decrease additional patient risk due to electricity shortage. Until LMICs have hospitals with near 100% reliable electricity grid systems, this is the reality of the health care system and new procedures should be in place based on additional risks to patients. The next subsection will calculate the grid unreliability versus percentage of medical procedures dependent on electricity (percentage of high impact, medium impact and low impact).

## 0.7 Details for Risk Matrix Results for the Four EHS-Types

The next subsection shows the detailed matrix plot for additional patients risks for one of the four health care facilities' energy systems discussed previously.

### 0.7.1 EHS-Type 1 in Region 1

Additional patient risks can be modeled for all four of the discussed healthcare facilities. The authors chose to exemplify the solar-powered facility in Iraq. This facility is a solar panel system with 20% energy capacity shortage. It illustrates the difficulty with powerload monitoring during the main(active) part of the day and the dependency on weather. Figure S8 shows the energy system failures on the top in red as well as the additional patient risk matrix calculations.

**S9a Fig. Additional Risk Matrix associated with a Solar Energy System in Iraq. The left three plots are associated with High Impact Medical Procedure grouping (max-top; mean-middle; min-bottom) whereas the right three plots are associated with the Low Impact Medical Procedure grouping (max-top; mean-middle; min- bottom).**

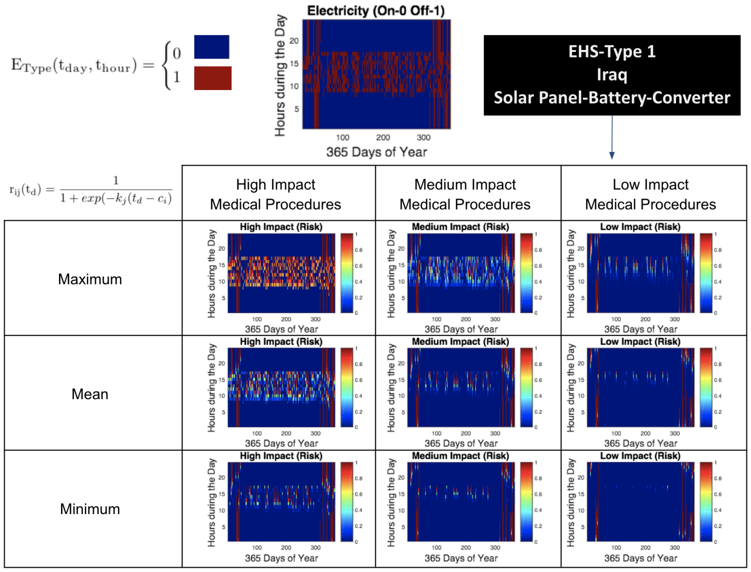


Yearly chance of death requires deleting all medical events that were not initiated because of a failure event. This may be during a day here the facility did not have any electricity all day. It is modeled as a postponement, which represents the reality in medical facilities in LMICs. Thus, the only medical procedures and, therefore, additional risks considered are when the electricity is on, and later fails.

**S9b Fig. Additional Risk Matrix associated with a Solar Energy System in Iraq after deleting the procedures that would have been postponed. The left three plots are associated with High Impact Medical Procedure grouping (max-top; mean-middle; min-bottom) whereas the right three plots are associated with the Low Impact Medical Procedure grouping (max-top; mean-middle; min- bottom).**

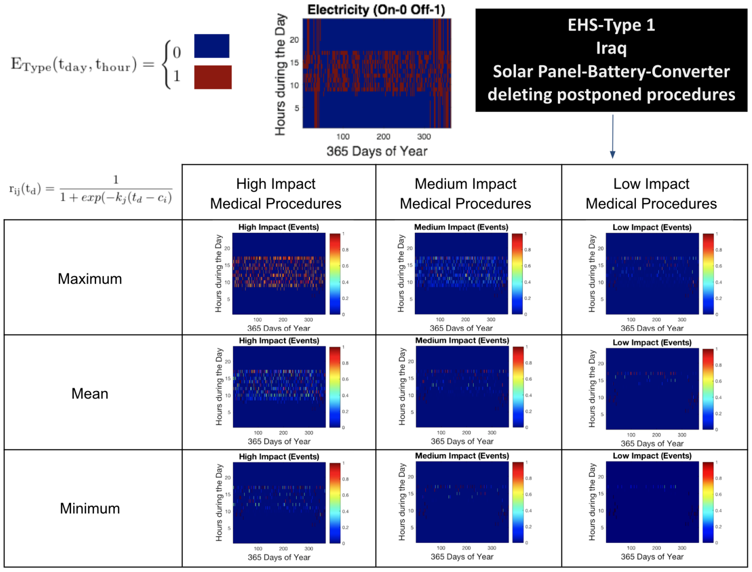


Figure S9b highlights clearly that the effect of power load depleting batteries during the day. From 9 am until 6pm for the entire year, the electricity is not safe for high impact medical procedures. If medical professionals knew how much energy was required for a medical procedure (say 10,000 kWh) and they knew how much energy was in the

battery (say 11,000 kWh), then they could decide to do the medical procedure, or to postpone it. Currently, there is no way of doing so, merely estimations from previous experience.

To calculate the additional patient risks in a year, in terms of patient deaths per 1000 patients, the authors averaged the hour-by-hour additional patient risks throughout the year for all nine risk matrices to create Figure S10.

**S9c Fig. The number of patient deaths, per 1,000, over the entire year (averaged over all electricity events) on the left is small compared to the right box plots for those who experience a failure (averaged only electricity failure events) during a medical procedure.**

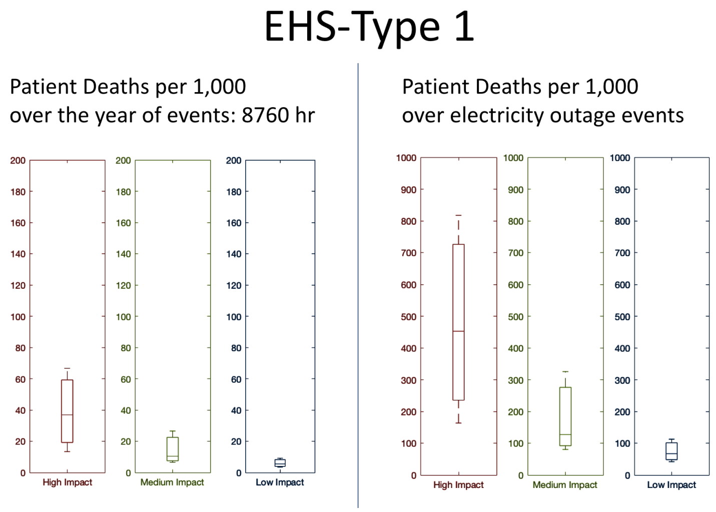


The left three box plots are the risks for patients with and without electricity whereas the right three box plots shows the risks only for patients who experience electricity failures. The risks for patients who experience electricity failures is much higher as expected.

These additional patient risks due to (1) impact on medical procedure due to electricity failure and (2) specific type of healthcare energy system are used to calculate a small, medium, and large health care facility number of deaths and the calculated Value of Statistical Life lost due to this amount of electricity failure ($/kWh).

### 0.7.2 EHS-Type 2 in Region 2

This health care facility is interesting because it is a hydroelectric system with 11% energy capacity shortage which illustrates the difficulty with dependence on the rainy season. Figure S10 shows the energy system failures on the top in red as well as the additional patient risk matrix calculations below. The figure seems to indicate that no one would want this energy system, but it offers electricity half the time to a health care facility with no electricity that performs medical procedures with low impact due to electricity failure. Like the solar only option, the medical procedures with low impact due to electricity have less additional patient risk to patients the majority of the time when the water is running.

**S10a Fig. Additional Risk Matrix associated with a Hydroelectric Energy System in Ghana. The left three plots are associated with High Impact Medical Procedure grouping (max-top; mean-middle; min-bottom) whereas the right three plots are associated with the Low Impact Medical Procedure**

grouping (max-top; mean-middle; min- bottom).

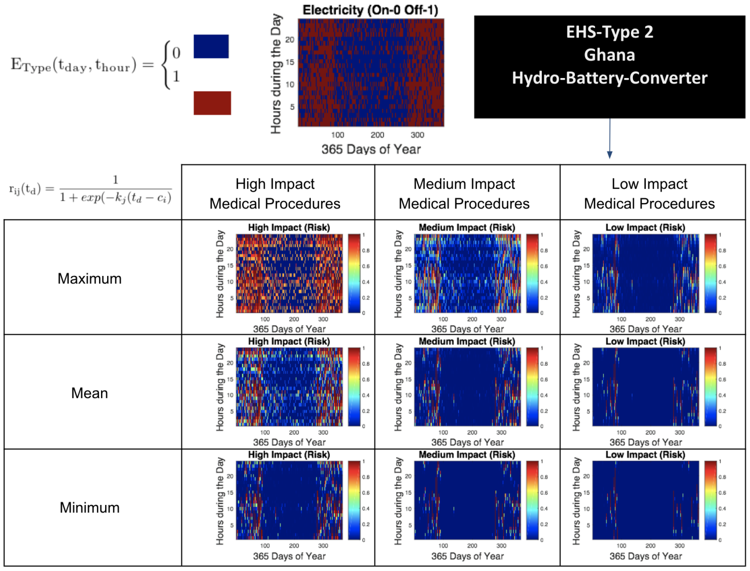


Yearly chance of death requires deleting all medical events that were not initiated because of a failure event. This may be during a day here the facility did not have any electricity all day. It is modeled as a postponement, which represents the reality in medical facilities in LMICs. Thus, the only medical procedures and, therefore, additional risks considered are when the electricity is on, and later fails.

**S10b Fig. Additional Risk Matrix associated with a Hydroelectric Energy System in Ghana after deleting the procedures that would have been postponed. The left three plots are associated with High Impact Medical Procedure grouping (max-top; mean-middle; min-bottom) whereas the right three plots are associated with the Low Impact Medical Procedure grouping (max-top; mean-middle; min- bottom).**

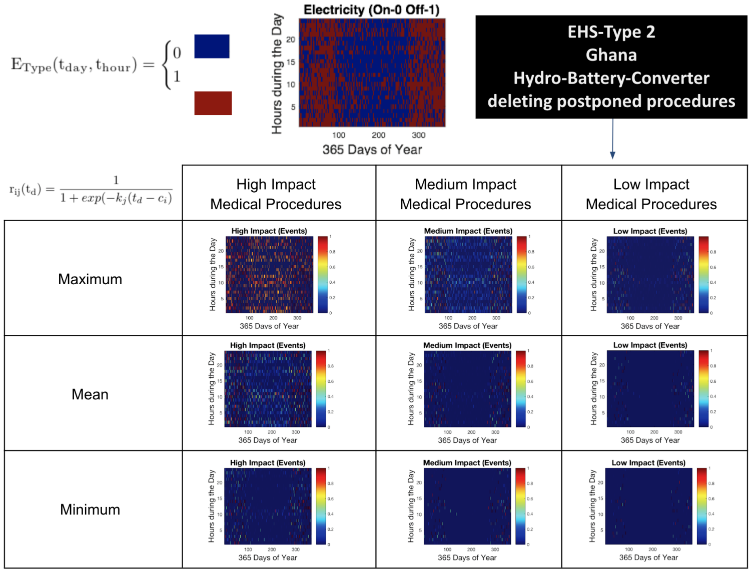


To calculate the additional patient risks in a year, in terms of patient deaths per 1000 patients, the authors averaged the hour-by-hour additional patient risks throughout the year for all nine risk matrices to create Figure S10.

**S10c Fig. The number of patient deaths, per 1,000, over the entire year (averaged over all electricity events) on the left is small compared to the right box plots for those who experience a failure (averaged only electricity failure events) during a medical procedure.**

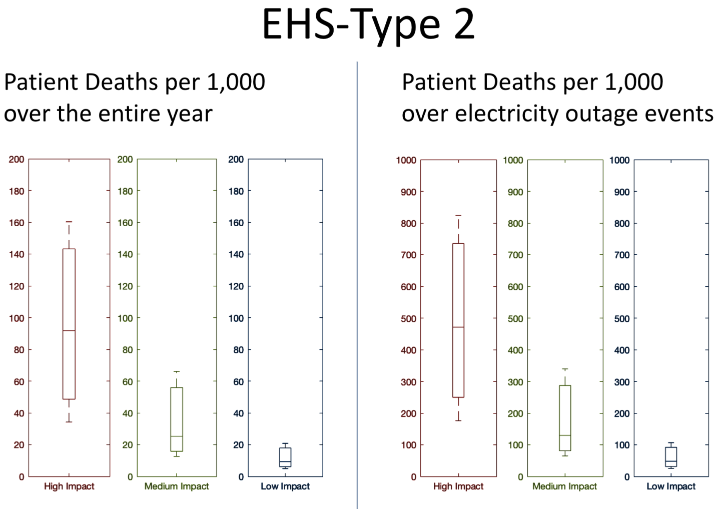


The left three box plots are the risks for patients with and without electricity whereas the right three box plots shows the risks only for patients who experience electricity failures. The risks for patients who experience electricity failures is much higher as expected.

These additional patient risks due to (1) impact on medical procedure due to electricity failure and (2) specific type of healthcare energy system are used to calculate a small, medium, and large health care facility number of deaths and the calculated Value of Statistical Life lost due to this amount of electricity failure ($/kWh).

### 0.7.3 EHS-Type 3 in Region 3

This health care facility is interesting because it is a hybridized energy system with 10% energy capacity shortage which highlights the benefits of hybridization. Figure S11 shows the energy system failures on the top in red as well as the additional patient risk matrix calculations below.

**S11a Fig. Additional Risk Matrix associated with a Hybridized Solar and Wind Energy System in Bangladesh. The left three plots are associated with High Impact Medical Procedure grouping (max-top; mean-middle; min-bottom) whereas the right three plots are associated with the Low Impact Medical Procedure grouping (max-top; mean-middle; min- bottom).**

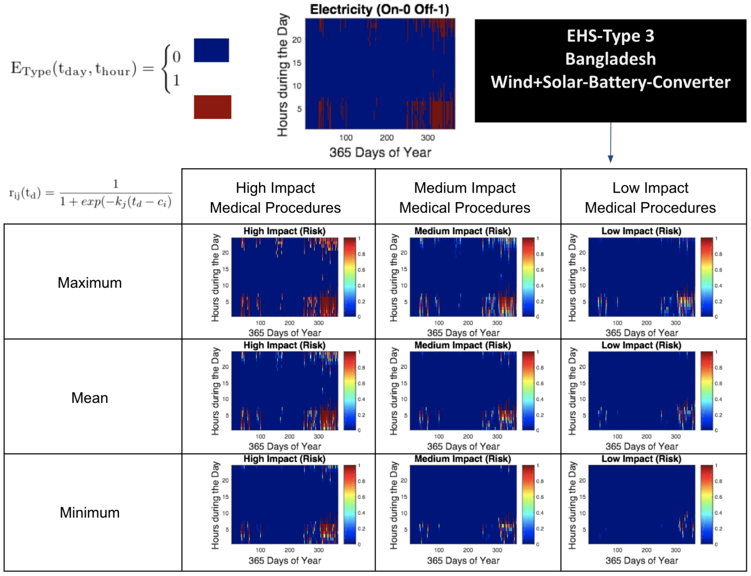


Yearly chance of death requires deleting all medical events that were not initiated because of a failure event. This may be during a day here the facility did not have any electricity all day. It is modeled as a postponement, which represents the reality in medical facilities in LMICs. Thus, the only medical procedures and, therefore, additional risks considered are when the electricity is on, and later fails.

**S11b Fig. Additional Risk Matrix associated with a Hybridized Solar and Wind Energy System in Bangladesh after deleting the procedures that would have been postponed. The left three plots are associated with High Impact Medical Procedure grouping (max-top; mean-middle; min-bottom) whereas the right three plots are associated with the Low Impact Medical Procedure grouping (max-top; mean-middle; min- bottom).**

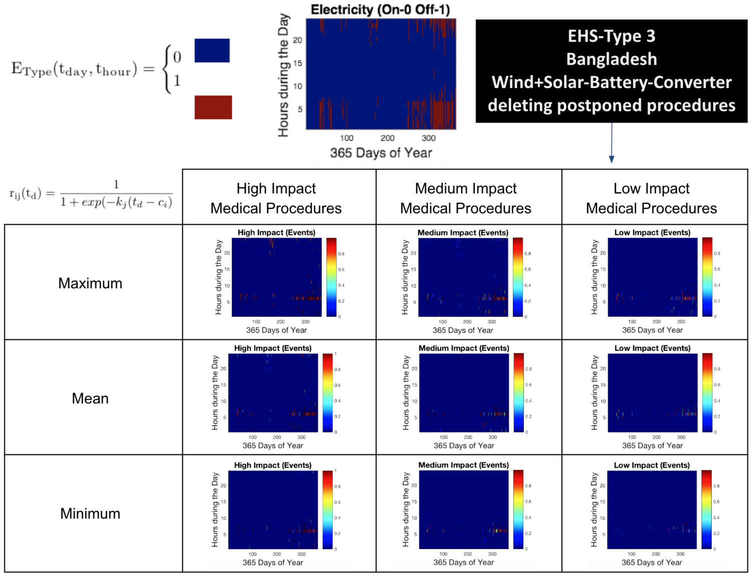


To calculate the additional patient risks in a year, in terms of patient deaths per 1000 patients, the authors averaged the hour-by-hour additional patient risks throughout the year for all nine risk matrices to create Figure S11.

**S11c Fig. The number of patient deaths, per 1,000, over the entire year (averaged over all electricity events) on the left is small compared to the right box plots for those who experience a failure (averaged only electricity failure events) during a medical procedure.**

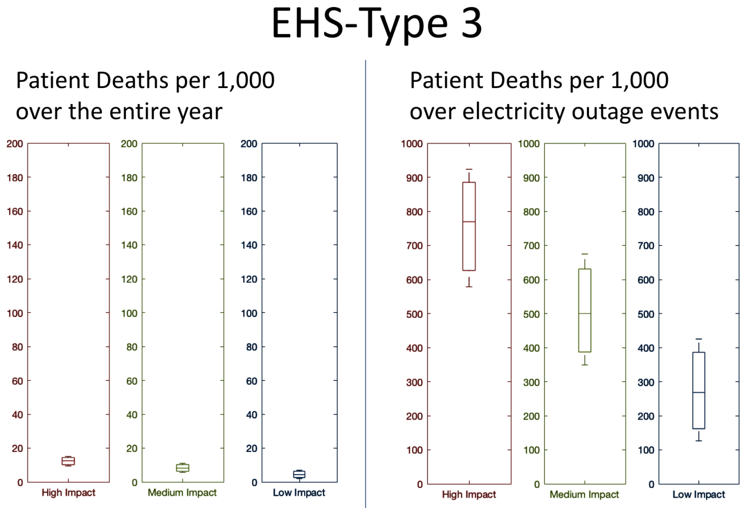


The left three box plots are the risks for patients with and without electricity whereas the right three box plots shows the risks only for patients who experience electricity failures. The risks for patients who experience electricity failures is much higher as expected.

These additional patient risks due to (1) impact on medical procedure due to electricity failure and (2) specific type of healthcare energy system are used to calculate a small, medium, and large health care facility number of deaths and the calculated Value of Statistical Life lost due to this amount of electricity failure ($/kWh).

### 0.7.4 EHS-Type 4 in Region 4

This health care facility is interesting because it is a centralized grid with backup diesel generator energy system with 4% energy capacity shortage which highlights the difficulties assuming that a backup diesel generator will take care of everything. Figure S12 shows the energy system failures on the top in red as well as the additional patient risk matrix calculations below.

**S12a Fig. Additional Risk Matrix associated with a Centralized Grid with Backup Diesel Generator Energy System in Uganda. The left three plots are associated with High Impact Medical Procedure grouping (max-top; mean-middle; min-bottom) whereas the right three plots are associated with the Low Impact Medical Procedure grouping (max-top; mean-middle; min- bottom).**

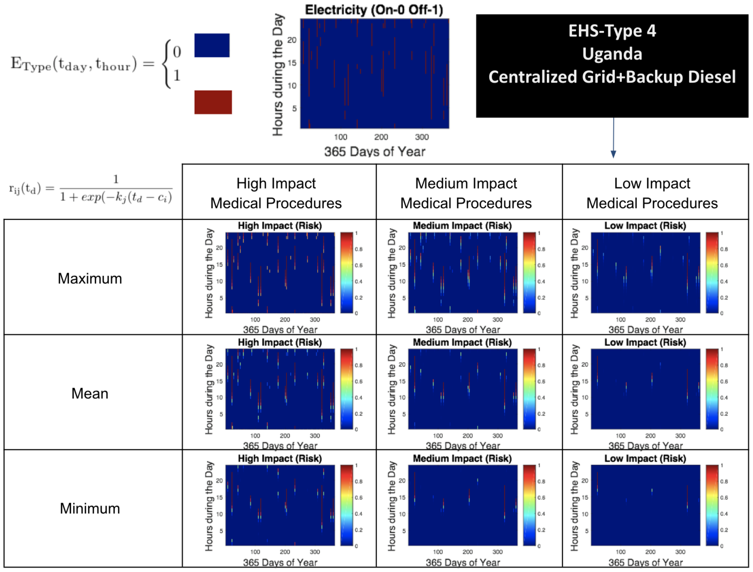


Yearly chance of death requires deleting all medical events that were not initiated because of a failure event. This may be during a day here the facility did not have any electricity all day. It is modeled as a postponement, which represents the reality in medical facilities in LMICs. Thus, the only medical procedures and, therefore, additional risks considered are when the electricity is on, and later fails.

**S12b Fig. Additional Risk Matrix associated with a Centralized Grid with Backup Diesel Generator Energy System in Uganda after deleting the procedures that would have been postponed. The left three plots are associated with High Impact Medical Procedure grouping (max-top; mean-middle; min-bottom) whereas the right three plots are associated with the Low Impact Medical Procedure grouping (max-top; mean-middle; min- bottom).**

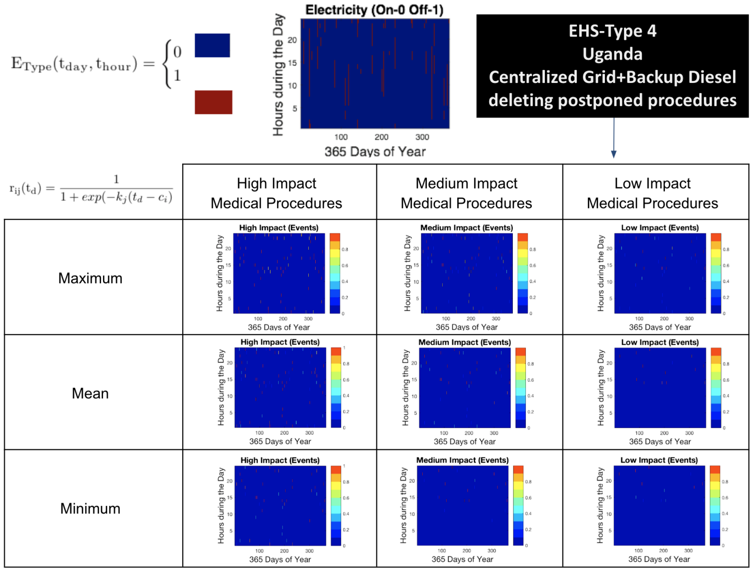


To calculate the additional patient risks in a year, in terms of patient deaths per 1000 patients, the authors averaged the hour-by-hour additional patient risks throughout the year for all nine risk matrices to create Figure S11.

**S12c Fig. The number of patient deaths, per 1,000, over the entire year (averaged over all electricity events) on the left is small compared to the right box plots for those who experience a failure (averaged only electricity failure events) during a medical procedure.**

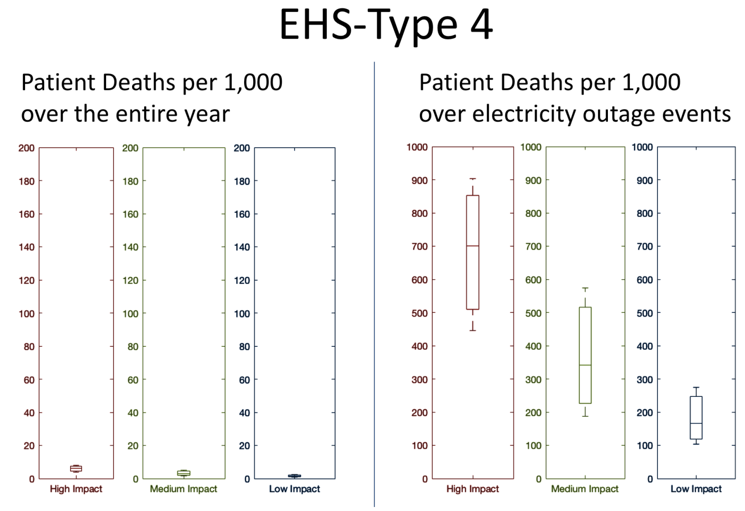


The left three box plots are the risks for patients with and without electricity whereas the right three box plots shows the risks only for patients who experience electricity failures. The risks for patients who experience electricity failures is much higher as expected.

These additional patient risks due to (1) impact on medical procedure due to electricity failure and (2) specific type of healthcare energy system are used to calculate a small, medium, and large health care facility number of deaths and the calculated Value of Statistical Life lost due to this amount of electricity failure ($/kWh).

### 0.7.5 Using Model to Verify a Claim of Number of Patient Deaths

Calculating patient deaths from the additional risk to patients due to electricity failure depends on many factors. The potential likelihood that Jinja Regional Hospital had 150 deaths in 6 months due solely to electricity failure, can be modeled and the verified.

**S13a Fig. The number of patient deaths modeled for Jinja Regional Referral hospital, based on a given procedure impact breakdown and based on these four EHS types.**

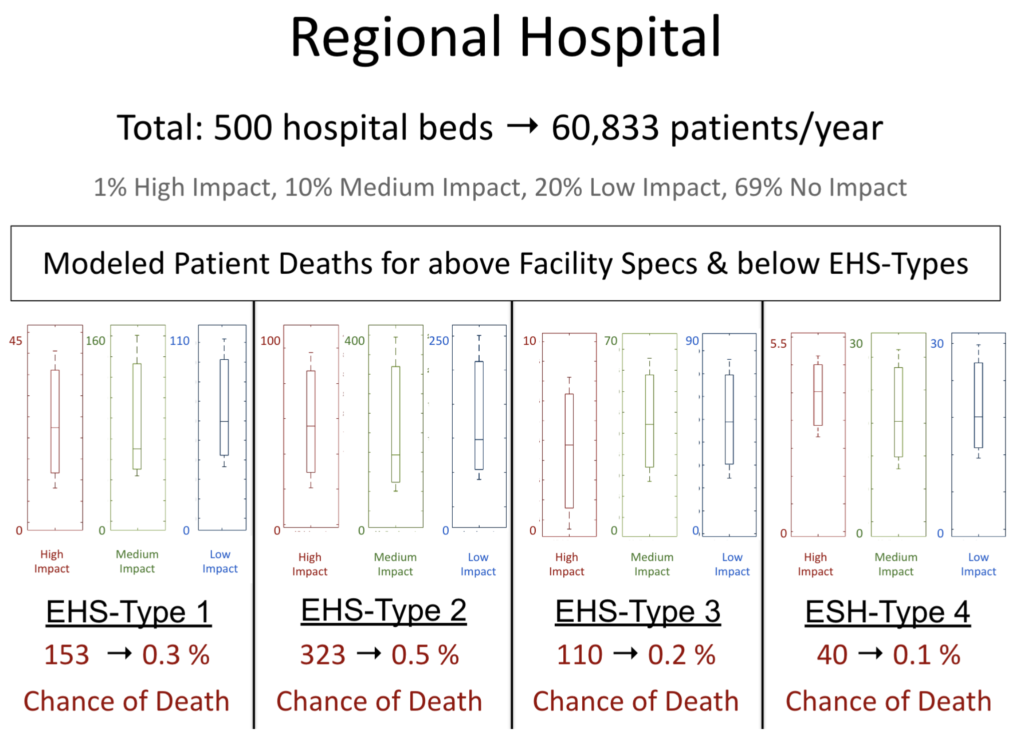


One might imagine that such a high death rate is associated with high failure rate to have this number of deaths in six months. However, this model result is from a grid+diesel energy system, potentially like the one in Jinja Regional Referral hospital, where the energy healthcare system only fails 4% of the time, randomly. The data and model also suggests that a larger percentage of medical procedures are in higher impact groups which seems reasonable for a regional referral hospital (where all medical procedures are divided into four risk impact groups - 45% high impact, 20% medium impact, 20% low impact, and 15% with no impact due to electricity failures).

**S13b Fig. The number of patient deaths modeled for Jinja Regional Referral hospital, based on a different procedure impact breakdown and based only on EHS-Type 4.**

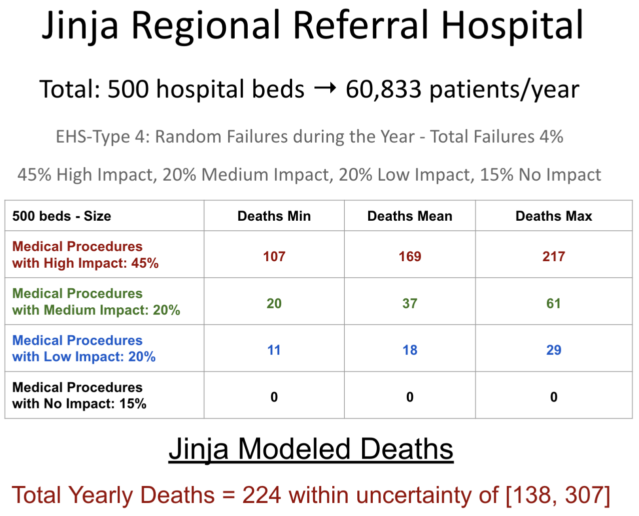


| 500 beds - Size | Deaths Min | Deaths Mean | Deaths Max |
|---|---|---|---|
| Medical Procedures with High Impact: 45% | 107 | 169 | 217 |
| Medical Procedures with Medium Impact: 20% | 20 | 37 | 61 |
| Medical Procedures with Low Impact: 20% | 11 | 18 | 29 |
| Medical Procedures with No Impact: 15% | 0 | 0 | 0 |

Figure S13b shows one permutation of possibilities based on real data of voltage and current taken for a year on a regional hospital in Uganda which was also defined as a EHS-Type 4. Given this information, 150 patients deaths in six months or 300 patients in a year is plausible. Specifically, this model calculates 224 deaths with a minimum of 138 deaths and a maximum of 307 deaths. More realistically, the grid at this hospital started to fail randomly at a higher rate with a lower number of medical procedures in the high and medium impact groups and a larger number of medical procedures in the low and no impact group. However, even at the low 4% failure rate, it is plausible that this many patients died due solely to electricity failures, especially considering surgeries that might have been started that were in an extremely high impact group which we did not consider (like brain and heart surgery where the patient would not survive for an entire hour without medical equipment requiring electricity).

#### 0.7.6 Scaling EHS-Type Models to Health Care Facilities

Because EHS-Types deal with failure patterns and not with power loads or specifical medical equipment, the health care communities can scale the failure pattern to their own health care facilities local data collection. Meaning that they can change the number of patients and percentages of their medical procedures into impact groups. This showcases the scalability of the model.

**S13c Fig. The number of patient deaths modeled for Small Health Care facility, based on medical procedures breakdown into impact groups and based on four EHS-Types.**

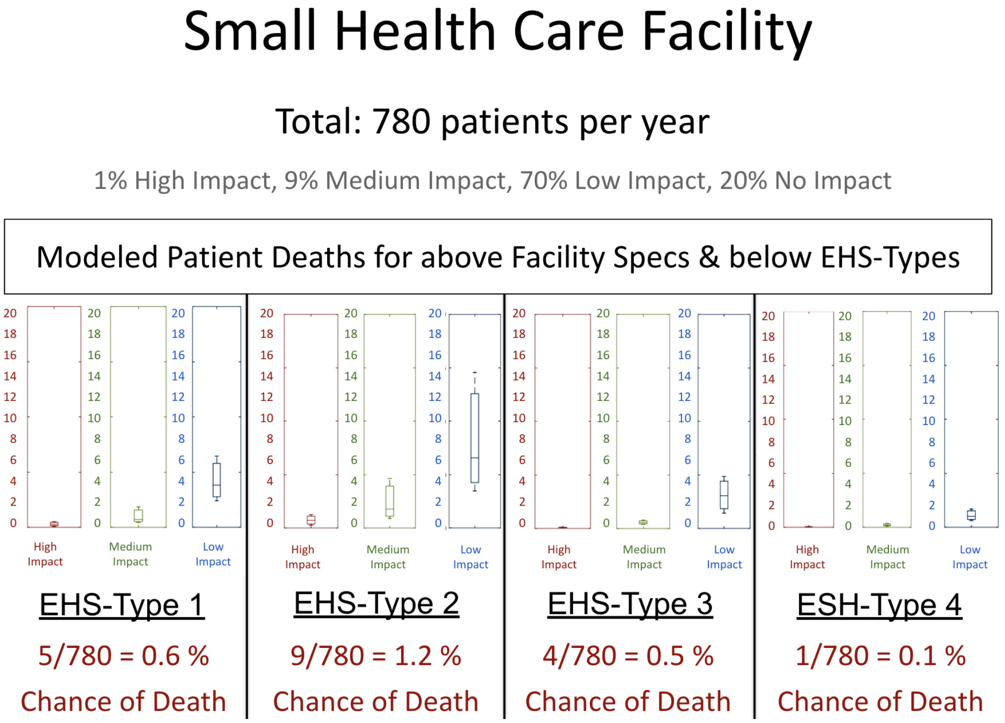


**S13d Fig. The number of patient deaths modeled for slightly Larger Health Care facility, based on medical procedures breakdown into impact groups and based on four EHS-Types.**

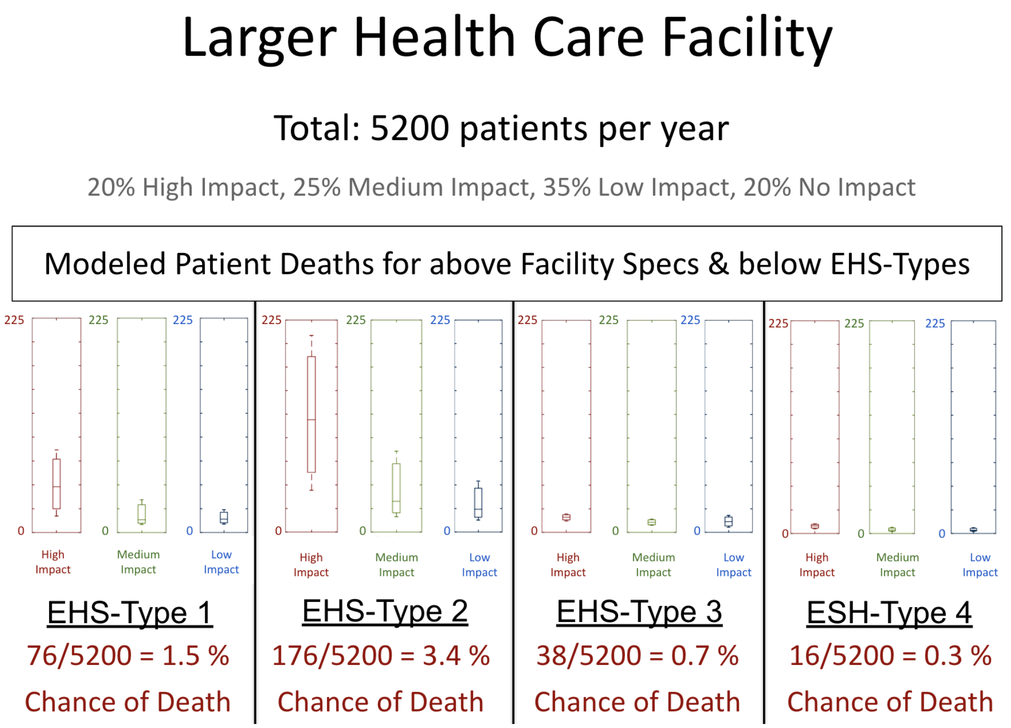


**S13e Fig. Comparison of the VSL/E results based on EHS-Type, Facility Sizes and Medical Procedures, and uncertainty in elasticities of countries.**

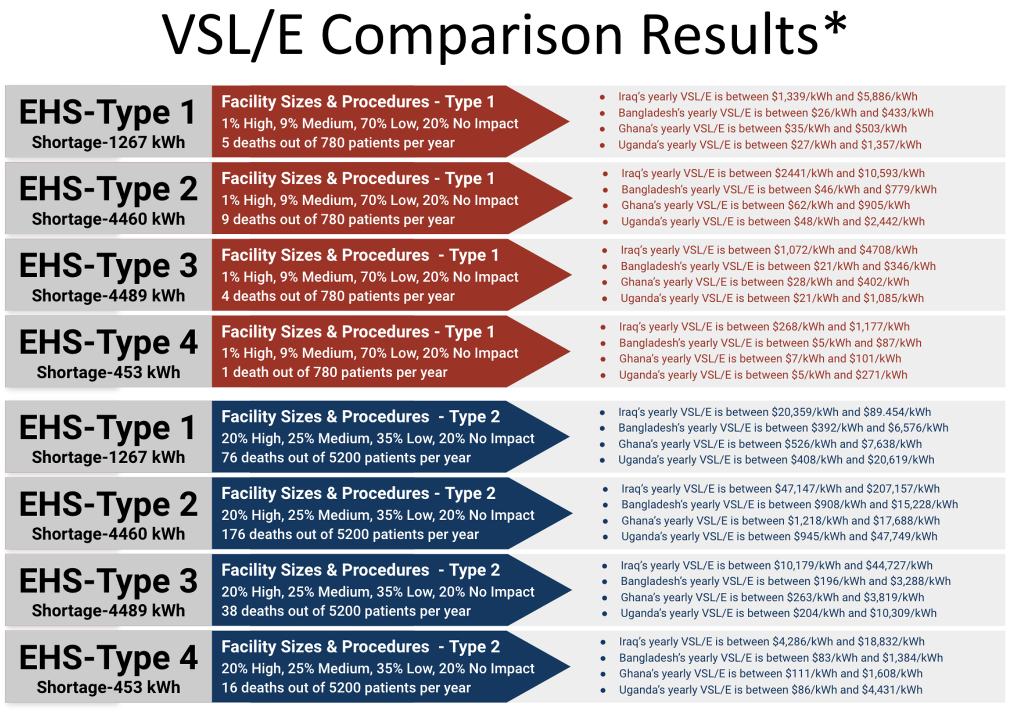


* VSL/E varies due to EHS-Types, Facility Sizes and Medical Procedures, and uncertainty in elasticities of countries.

As can be seen in comparing Figure S13C and S13d, the chance of death depends on the EHS-Types more than Facility Size and Medical Procedures versus Figure S13e

where the VSL/E depends more on Facility Size and Medical Procedures and less on EHS-Type. Unfortunately, box plots of risks to chance of death calculations as well as flow charts to VSL/E calculations are not clearly nor quickly understood which gave motivation for the next section.

### 0.7.7 Motivation to Simplify How this is Communicated

The authors of this study went back-and-forth for months and even years trying to create a way to communicate the energy designers knowledge with the medical community knowledge. A common language was needed and risk charts were common to both groups of professionals. This research paper is a result of this progression to EHS-Risk Charts. However, the ultimate goal is to communicate these results to a broader community and the flow chart of explanation looks like Figure S14a.

**S14a Fig. The foundation for the EHS-Risk Chart from EHS-Types to probabilistic surveys to percentages of medical procedures done based on impact types and overall size of facility**

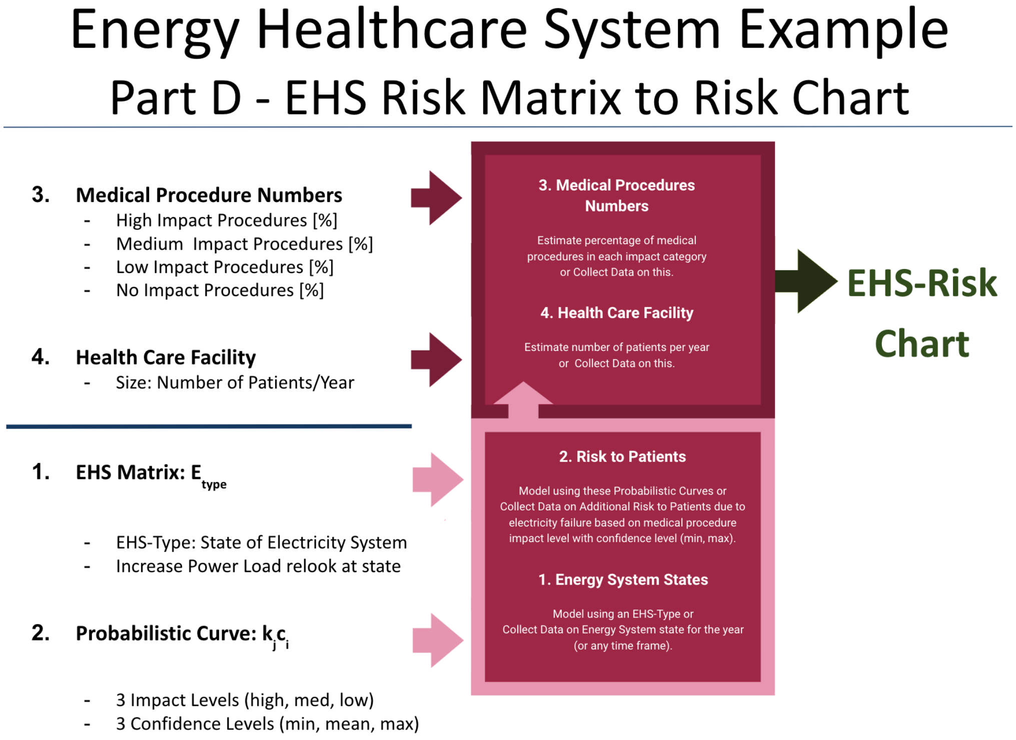


Given this information and knowing that many box plots and/or tables would be confusing and cumbersome to interpret quickly and act responsibly, the authors averaged the risk based on a day of the year and grouped the days into categories of severity of risk and likelihood of risk.

**S14b Fig. The basic EHS-Risk chart for EHS-Type 1, VSL-Iraq, and the labeled size of facility and percentages of medical procedure impact groups**

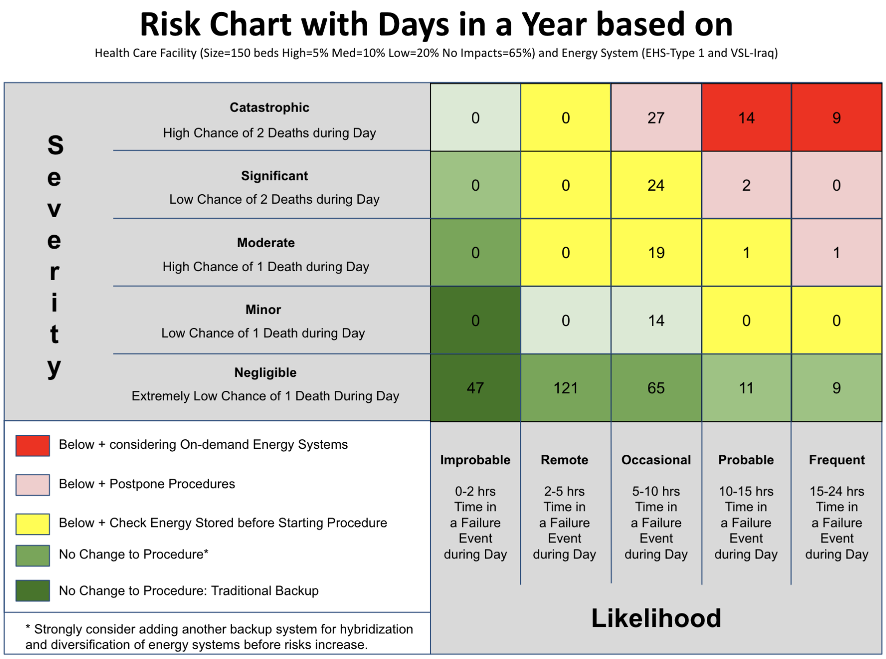


Other EHS-Risk Charts can be created and compared to the original one. This is especially interesting in increasing hybridization to the original EHS-Type, comparing different EHS types, or in changing the percentages of medical procedures in each impact level and comparing the outcomes. This should motivate the global medical community to collect data on patient outcomes when electricity fails.

**S14c Fig. The basic EHS-Risk chart for EHS-Type 4, VSL-Uganda, and the labeled size of facility and percentages of medical procedure impact groups**

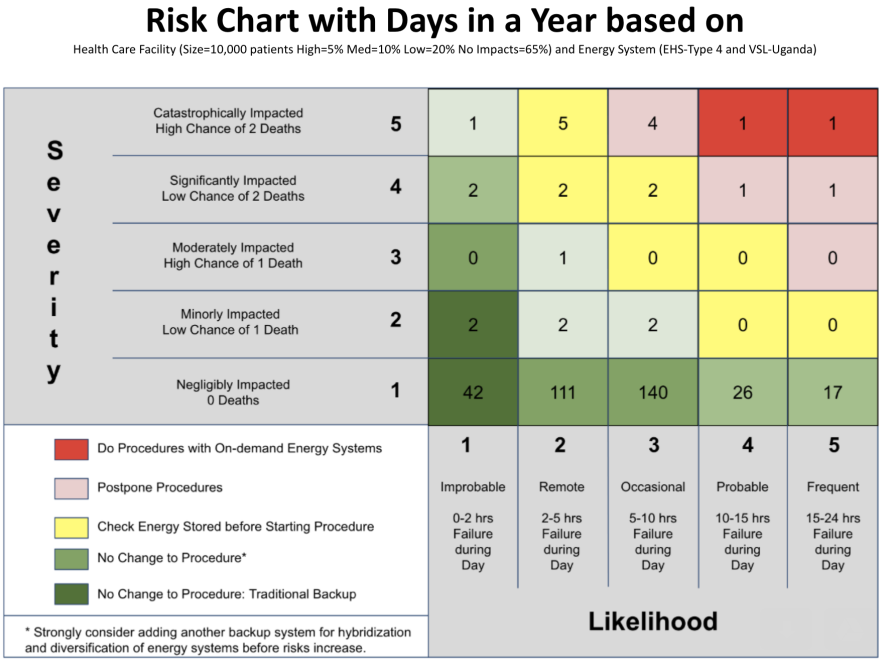


Now that the number of days are grouped, the VSL/E can be calculated for each

risk chart category. This can then be compared to the original costs or in considering new energy systems for hybridization among other things already discussed in the main paper recommendations.

**S14d Fig. The EHS-Risk chart with number of days facing this risk category and the hidden costs in terms of VSL/E values based on lives lost and energy shortage.**

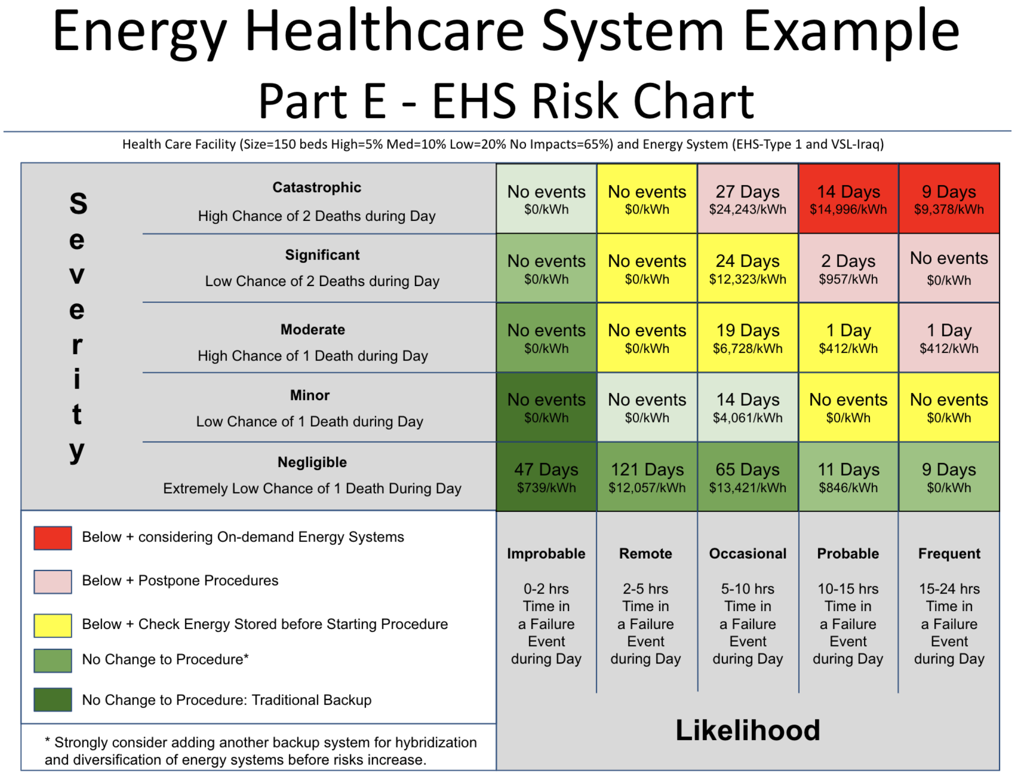


The most dramatic result are the VSL/E costs associated with this risk chart. These costs are orders of magnitude higher than the traditional levelized cost of electricity (LCOE) and this only considers one year. As power load increases, the failure rates can increase and these costs will grow. Likewise, as health care facilities add more backup systems for hybridization, the costs decrease.

——— End Edits Here for Now ———

## 0.8 Flexible Risk Charts in Making Medical Decisions under Uncertainty

A model is only as good as it is flexible under multiple scenarios and uncertainties. In our two parameter logistic model (c,k) with high, medium, and low impact types, the authors can easily, effectively and efficiently implement other 2 parameter probabilistic curves depending on data to estimate answers to these questions or anecdotal experience and uncertainties in these answers. However, the authors have not shown these results here. Upon request, these further details and investigations can be emailed to interested readers.

However, this particular methodology has interesting results with a serious note to critiques that based on Taylor's Theorem all of these mathematical functions have no statistical differences based on the 10 hours window considered and the level of uncertainties considered. This is a consequence of lack of data collection on additional

risk to patients after an electricity failure based on type of medical procedure and duration of failure. Once the data is collected, this ESH-Risk Chart methodology and model will adapt to the data (in terms of resultant energy failure matrices and probabilistic curves), will scale to the facility type (in terms of percentage of medical procedures placed in impact groups and total number of patients), and will ultimately be verifiable (calculate the VSL/E costs due to patient lives lost and the energy needed to provide electricity for that medical procedure).
