# Supplementary figures and images for: Health care during electricity failure: The hidden costs
Source: PLoS One. 2020 Nov 4;15(11):e0235760. doi: 10.1371/journal.pone.0235760 (PMC7641375; doi:10.1371/journal.pone.0235760)

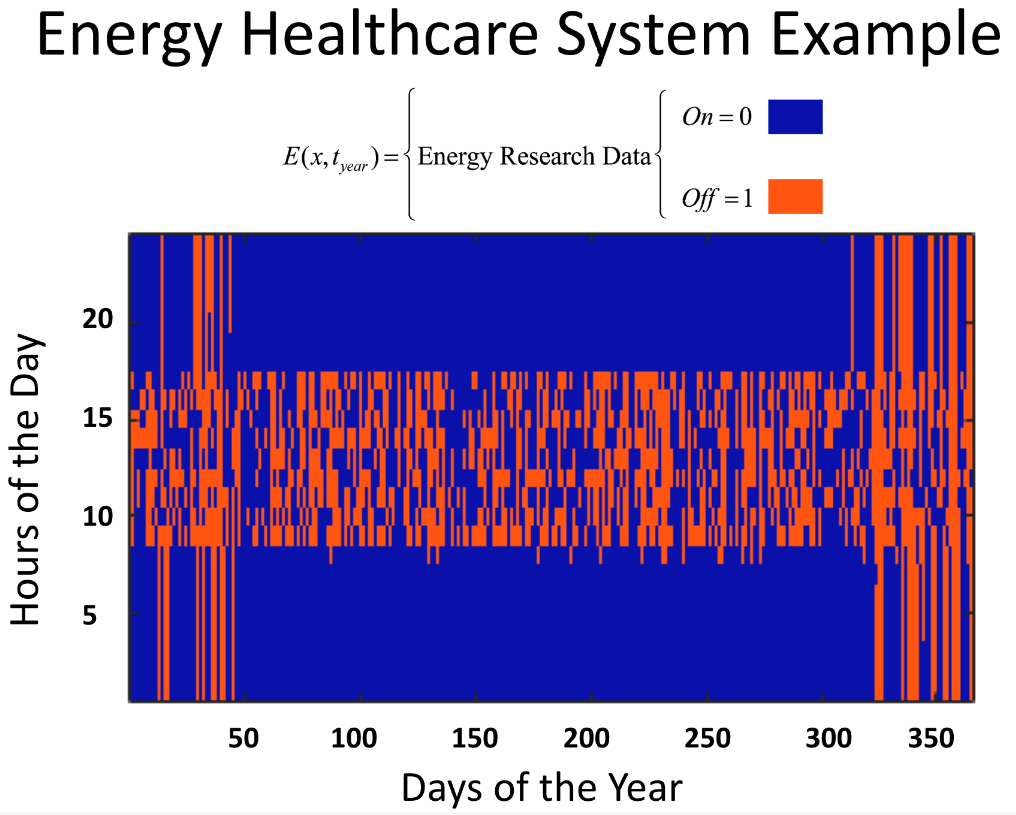

Supplement: S1 Fig — (TIFF) [file pone.0235760.s006.tiff]

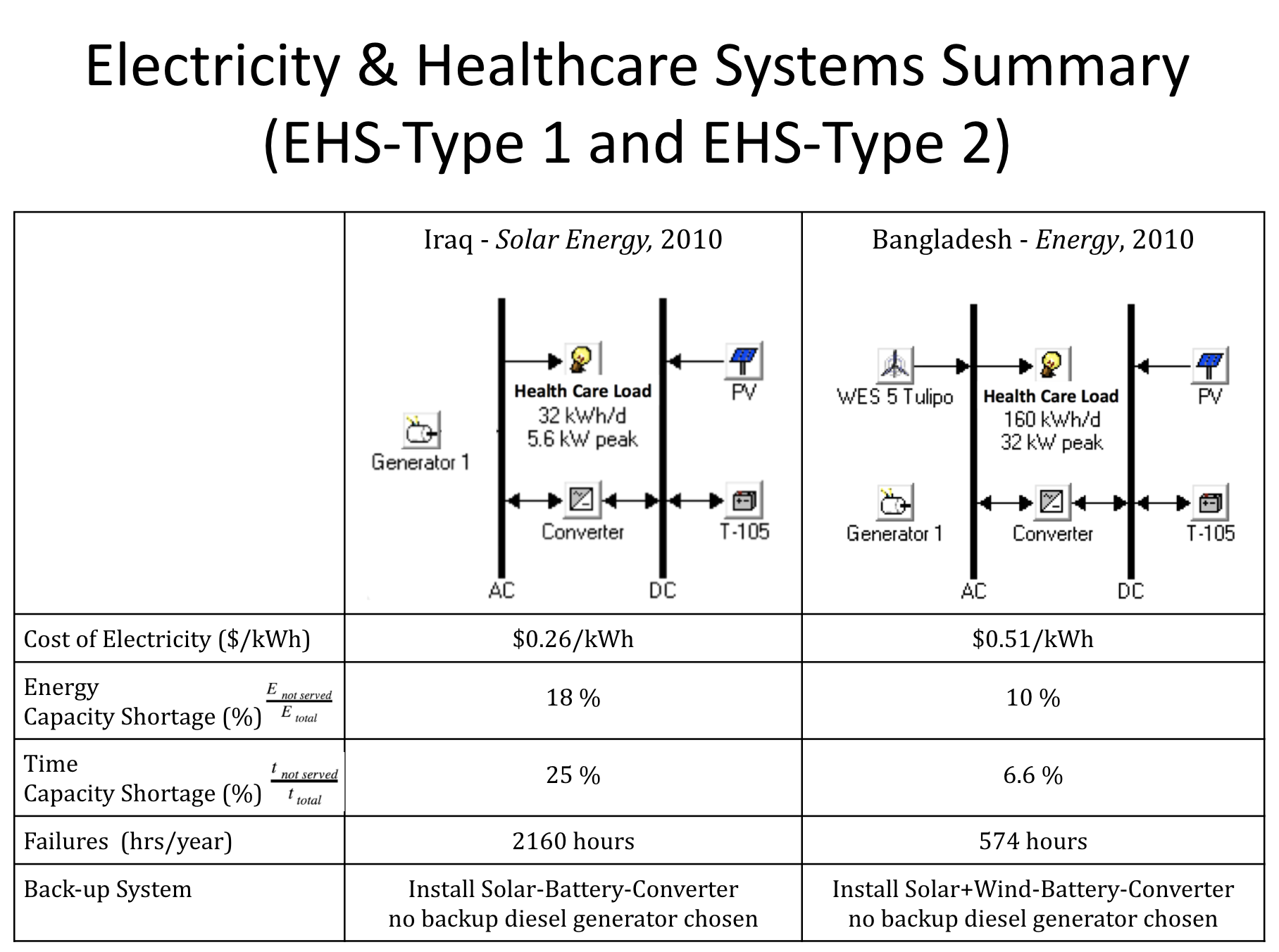

Supplement: S2 Fig — (TIFF) [file pone.0235760.s007.tiff]

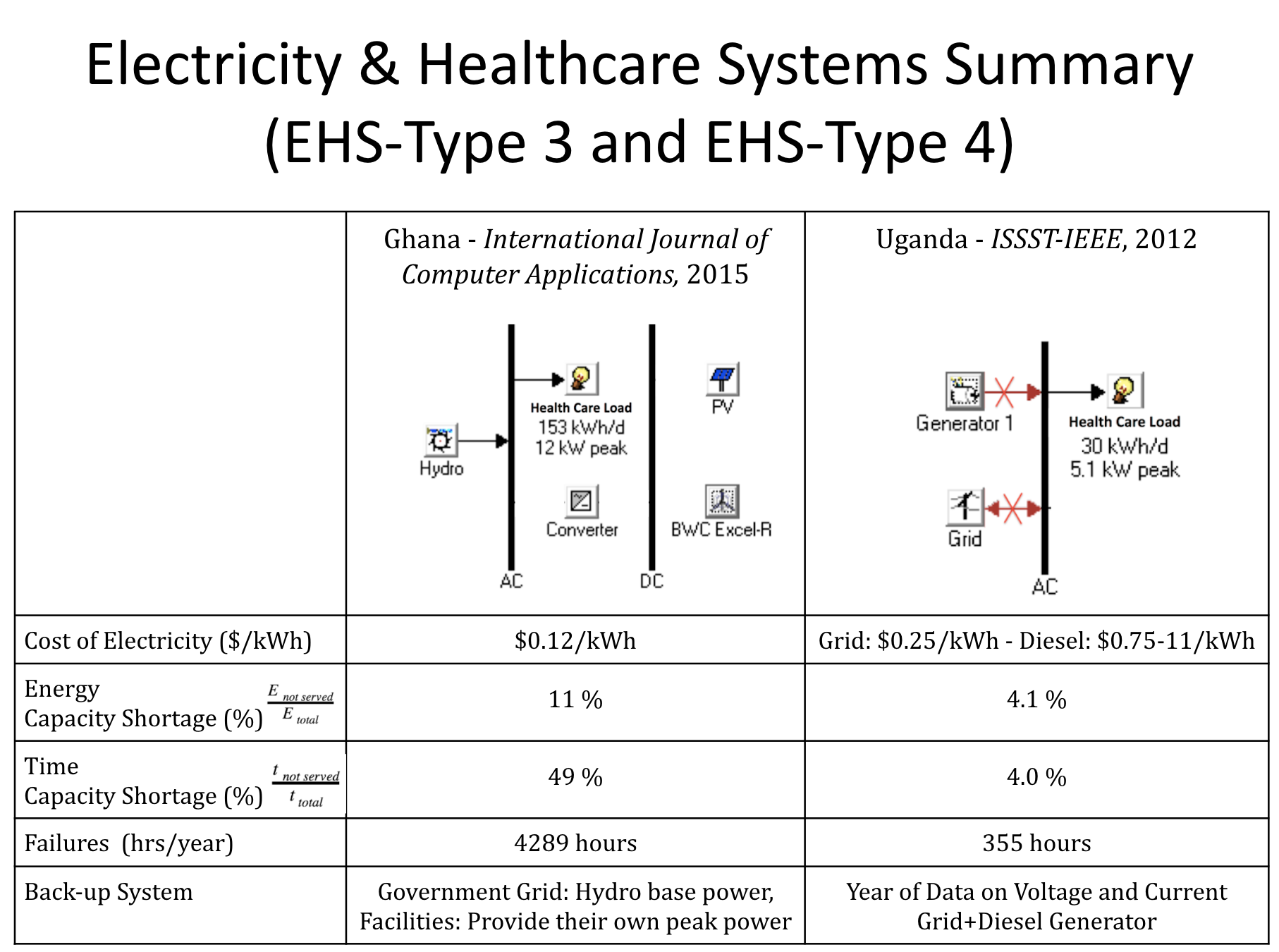

Supplement: S3 Fig — (TIFF) [file pone.0235760.s008.tiff]

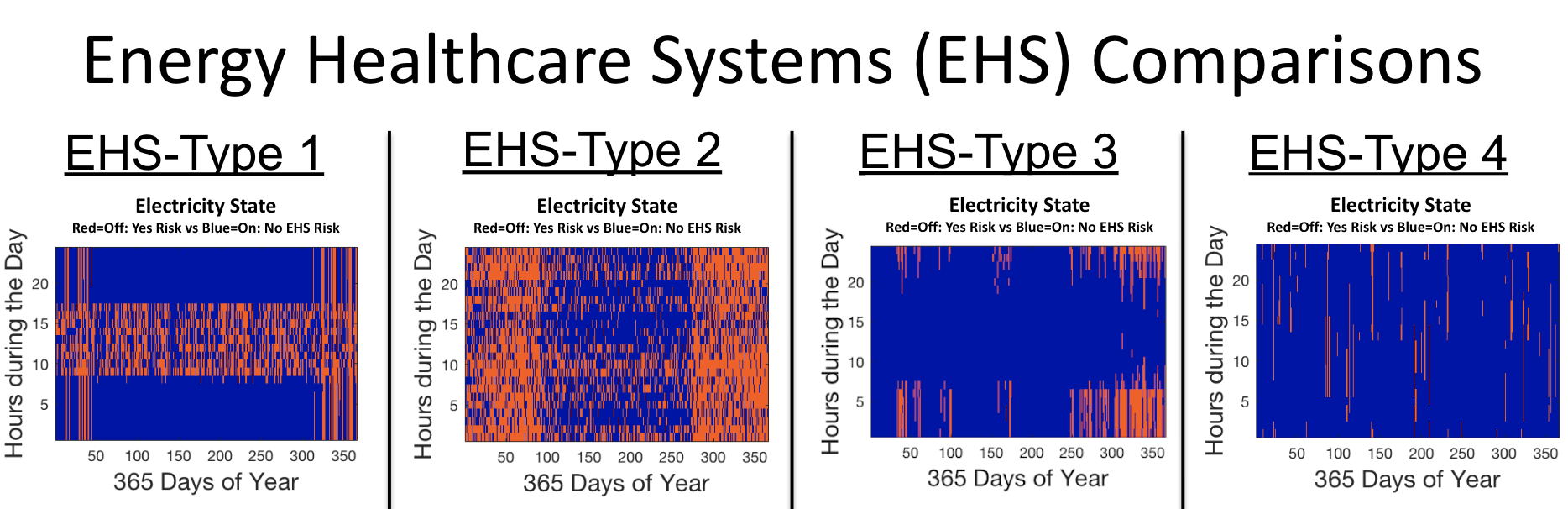

Supplement: S4 Fig — (TIFF) [file pone.0235760.s009.tiff]

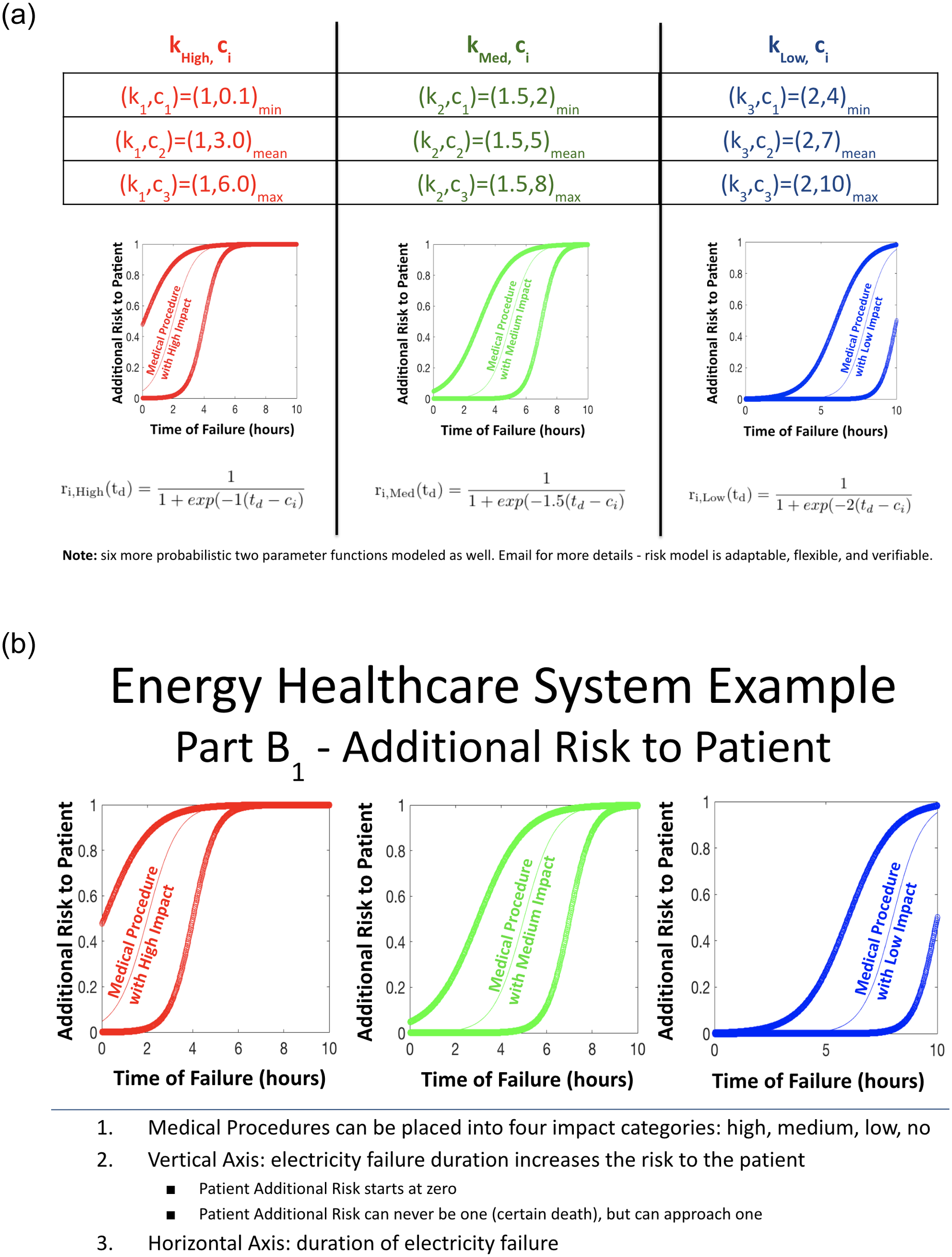

Supplement: S5 Fig — (TIFF) [file pone.0235760.s010.tiff]

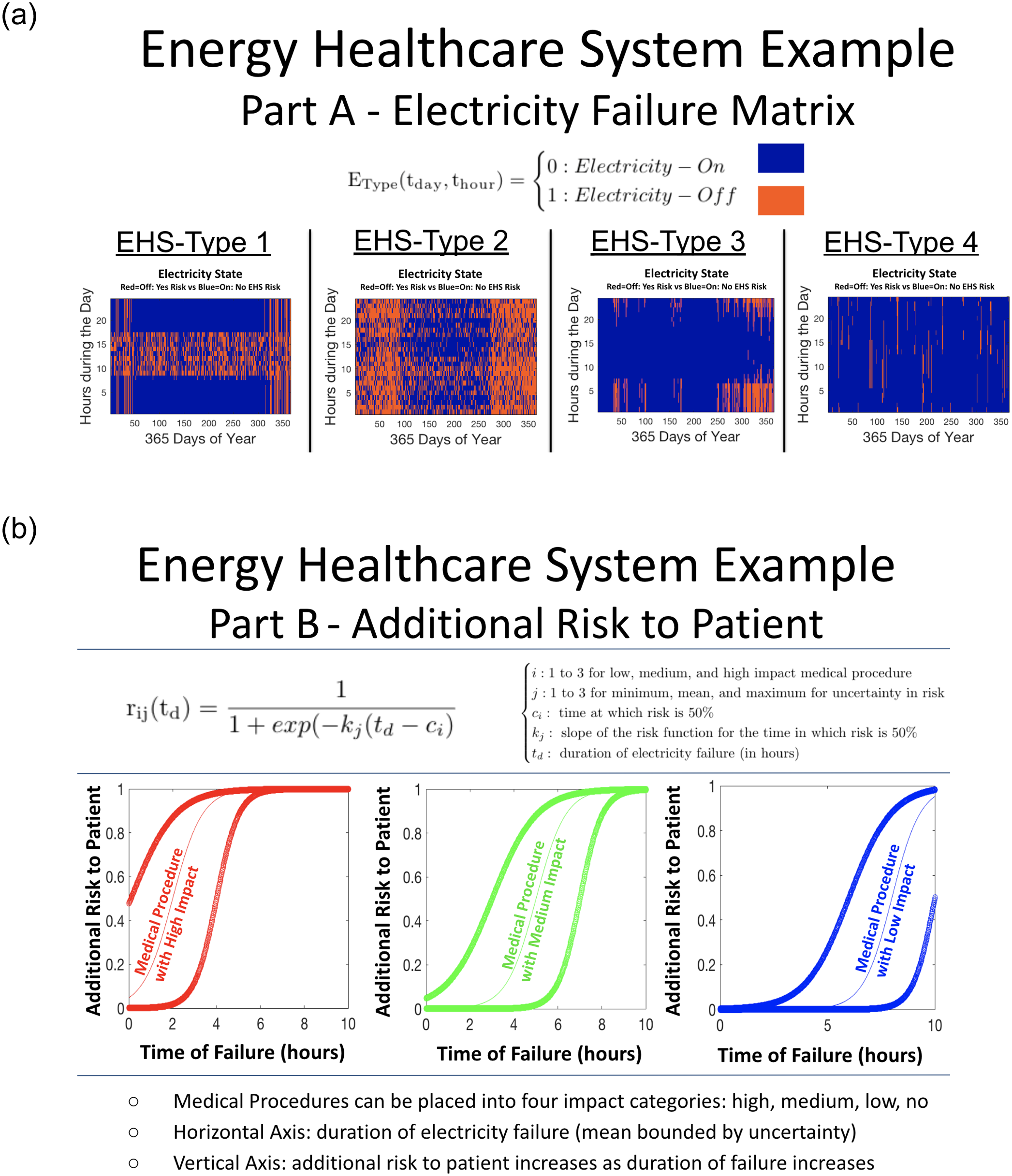

Supplement: S6 Fig — (TIFF) [file pone.0235760.s011.tiff]

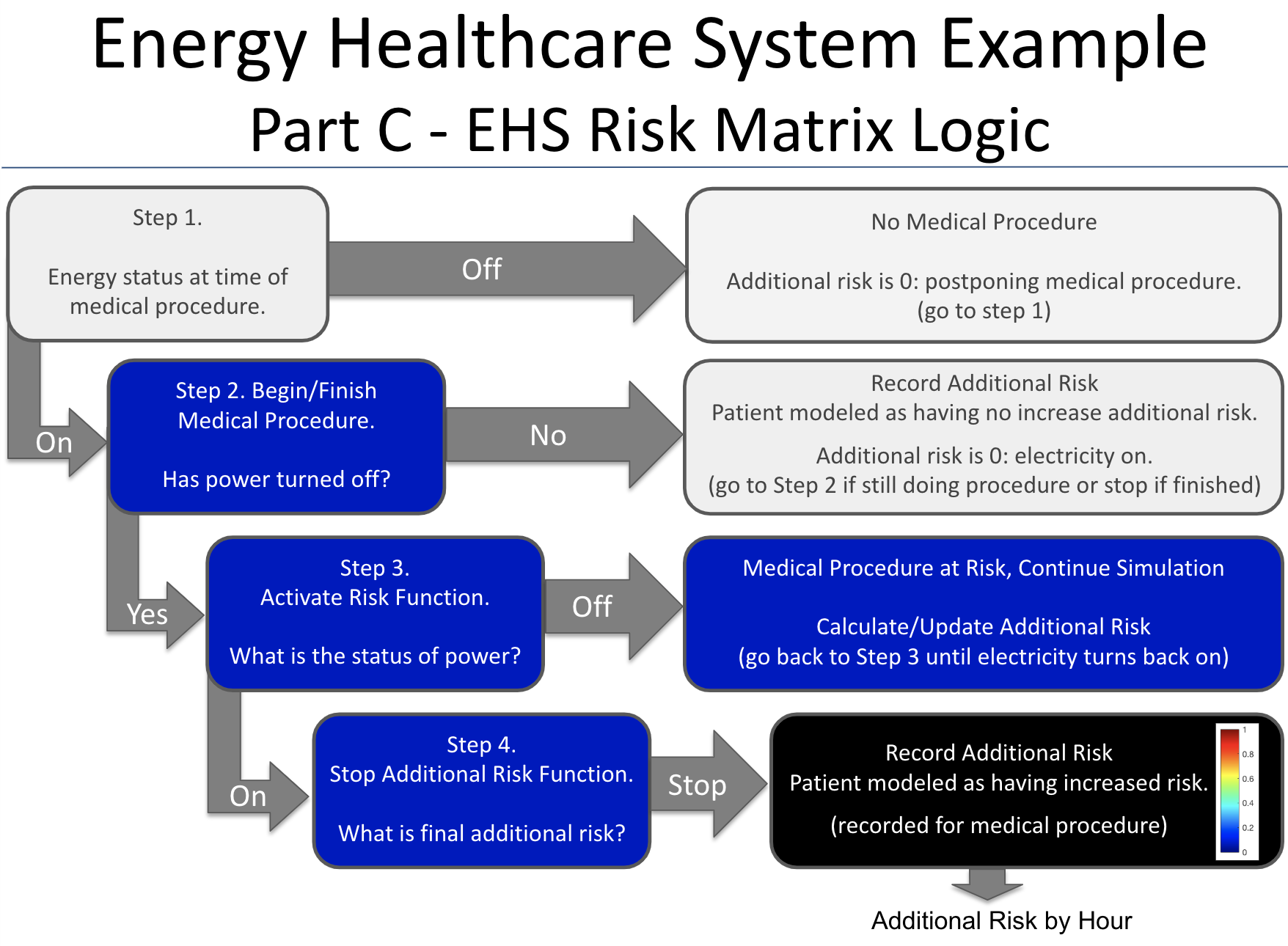

Supplement: S7 Fig — (TIFF) [file pone.0235760.s012.tiff]

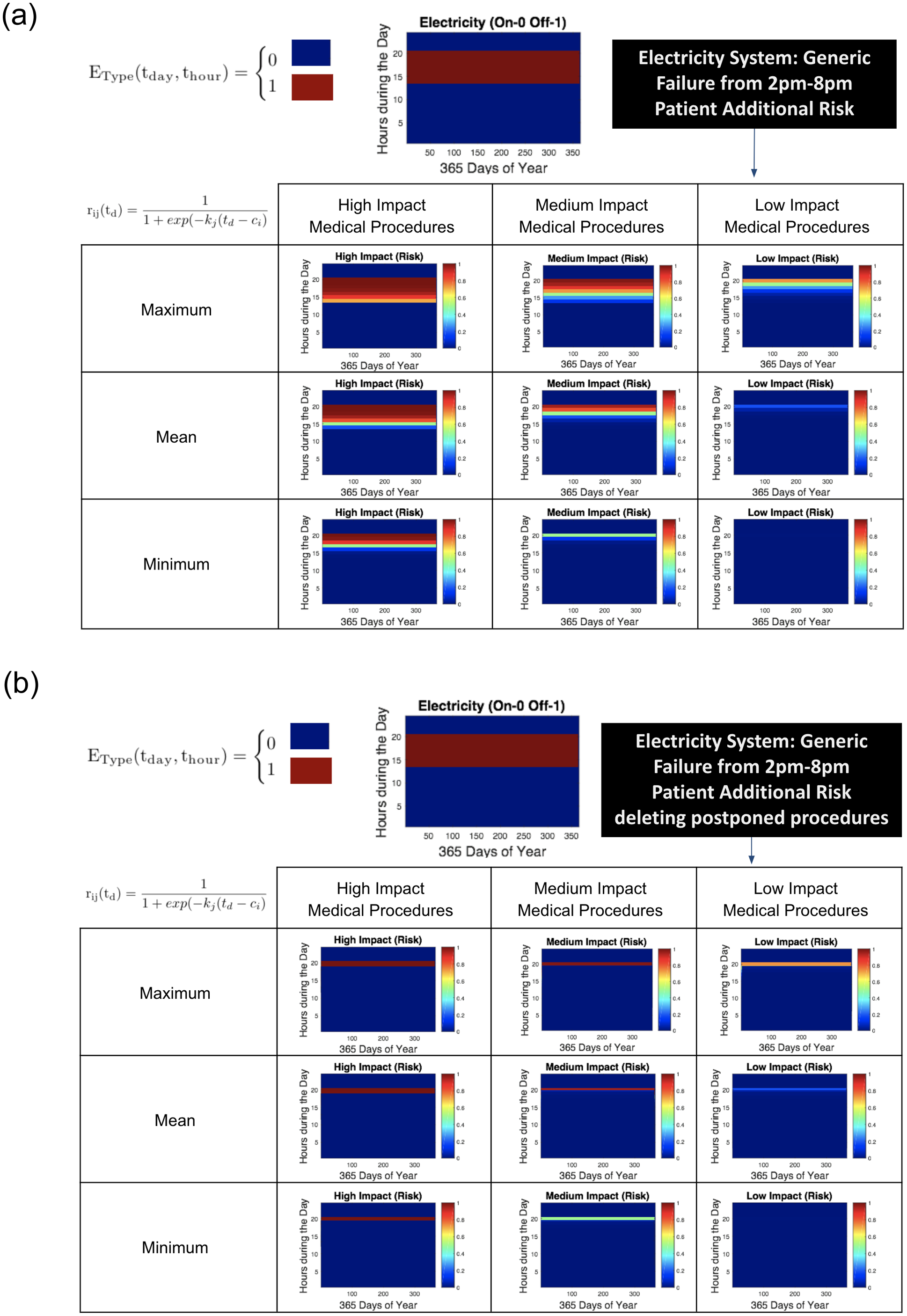

Supplement: S8 Fig — (TIFF) [file pone.0235760.s013.tiff]

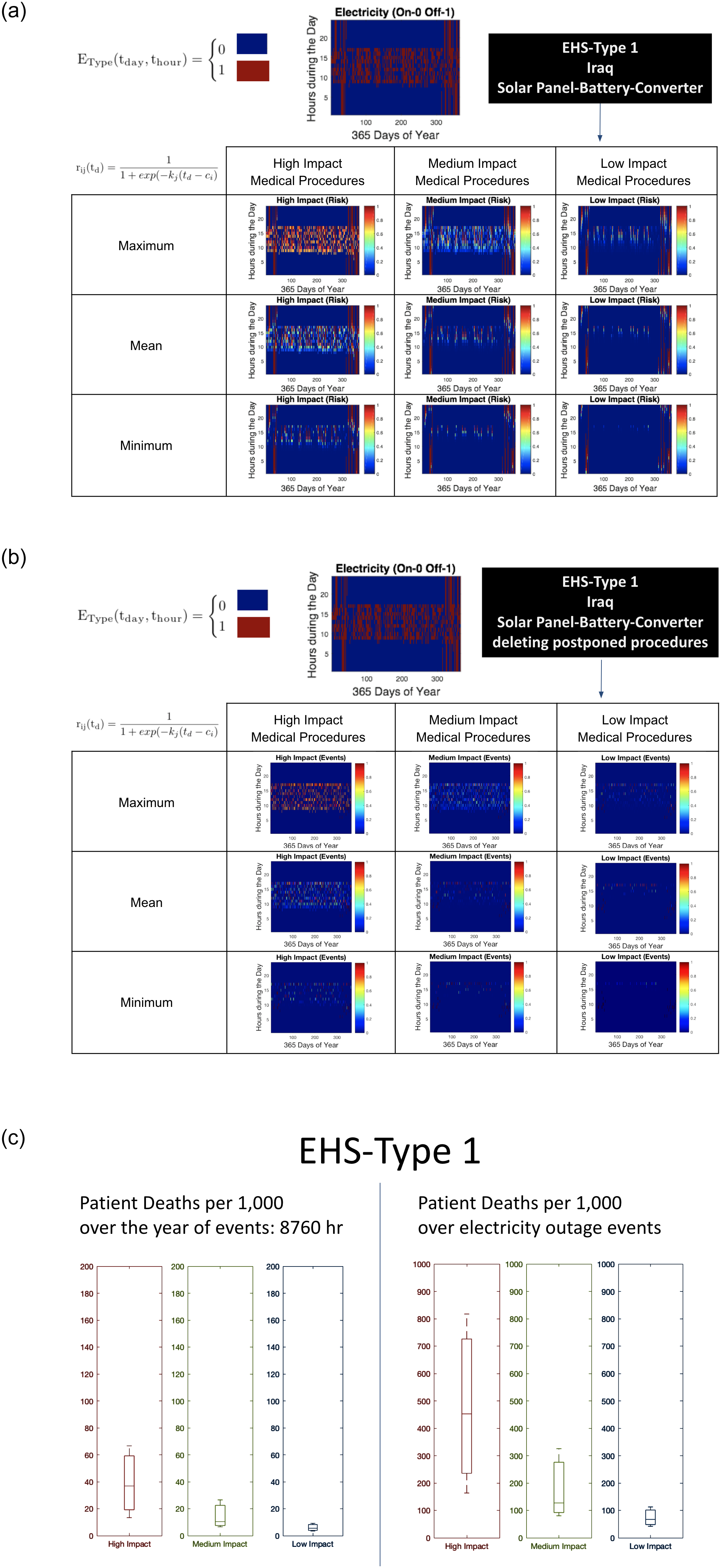

Supplement: S9 Fig — (TIFF) [file pone.0235760.s014.tiff]

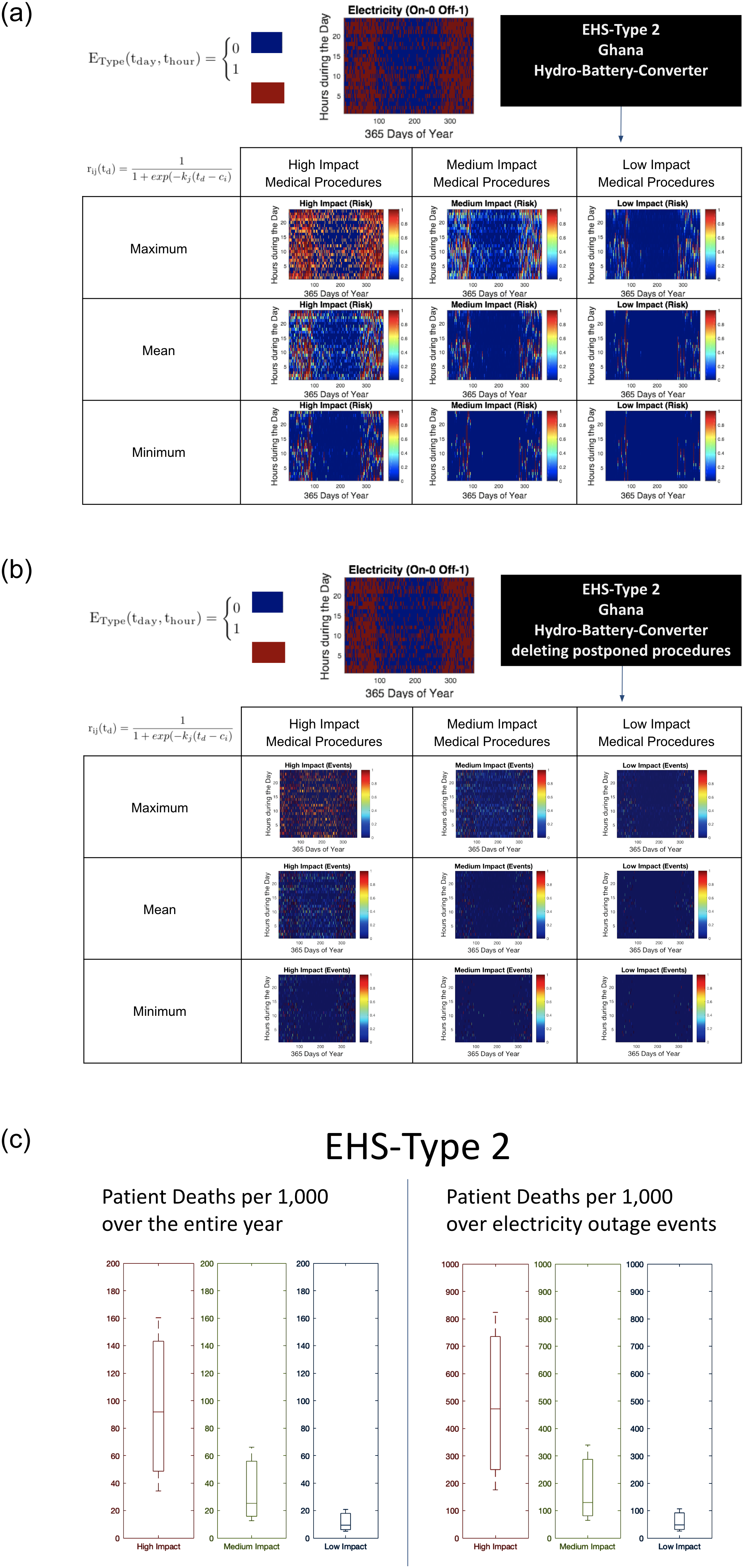

Supplement: S10 Fig — (TIFF) [file pone.0235760.s015.tiff]

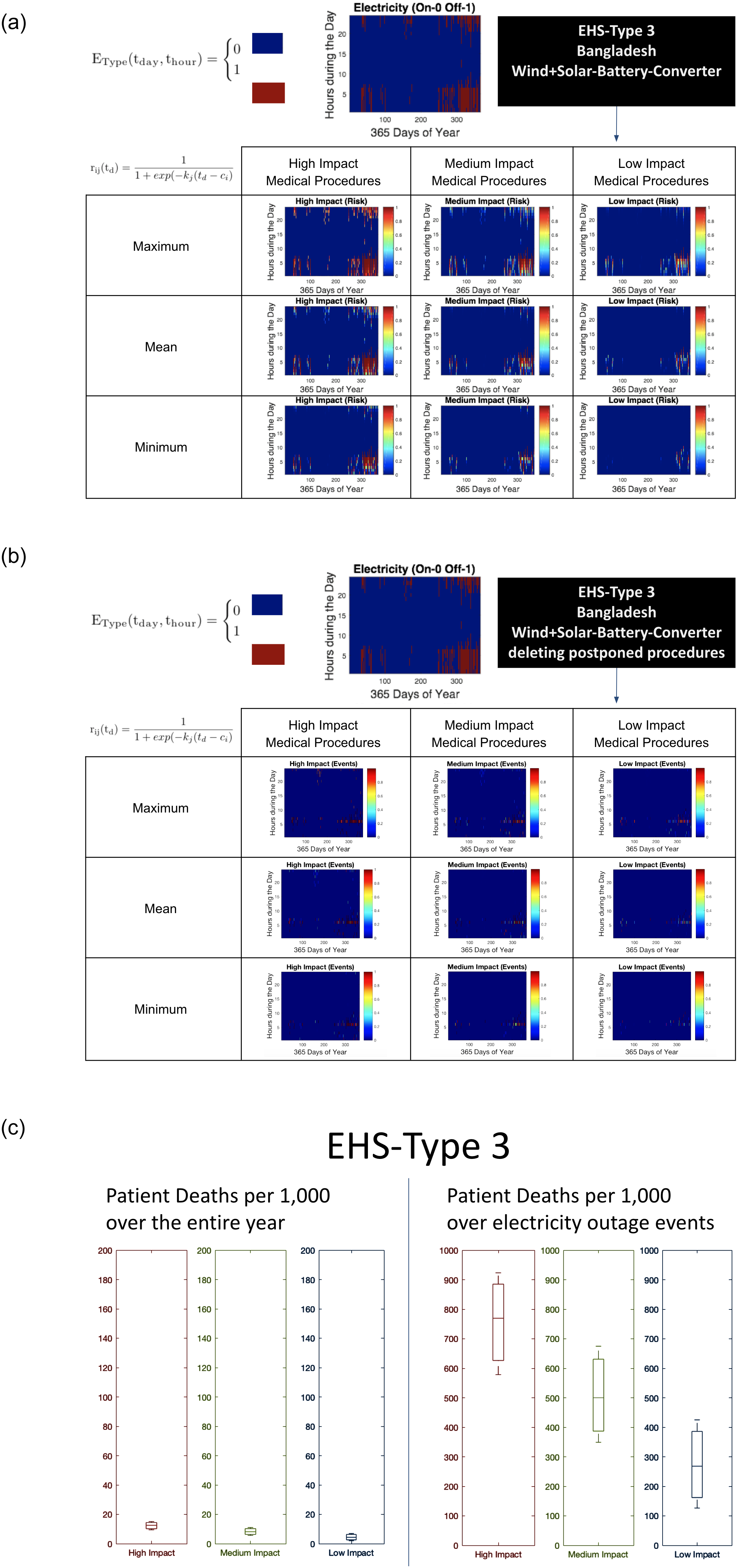

Supplement: S11 Fig — (TIFF) [file pone.0235760.s016.tiff]

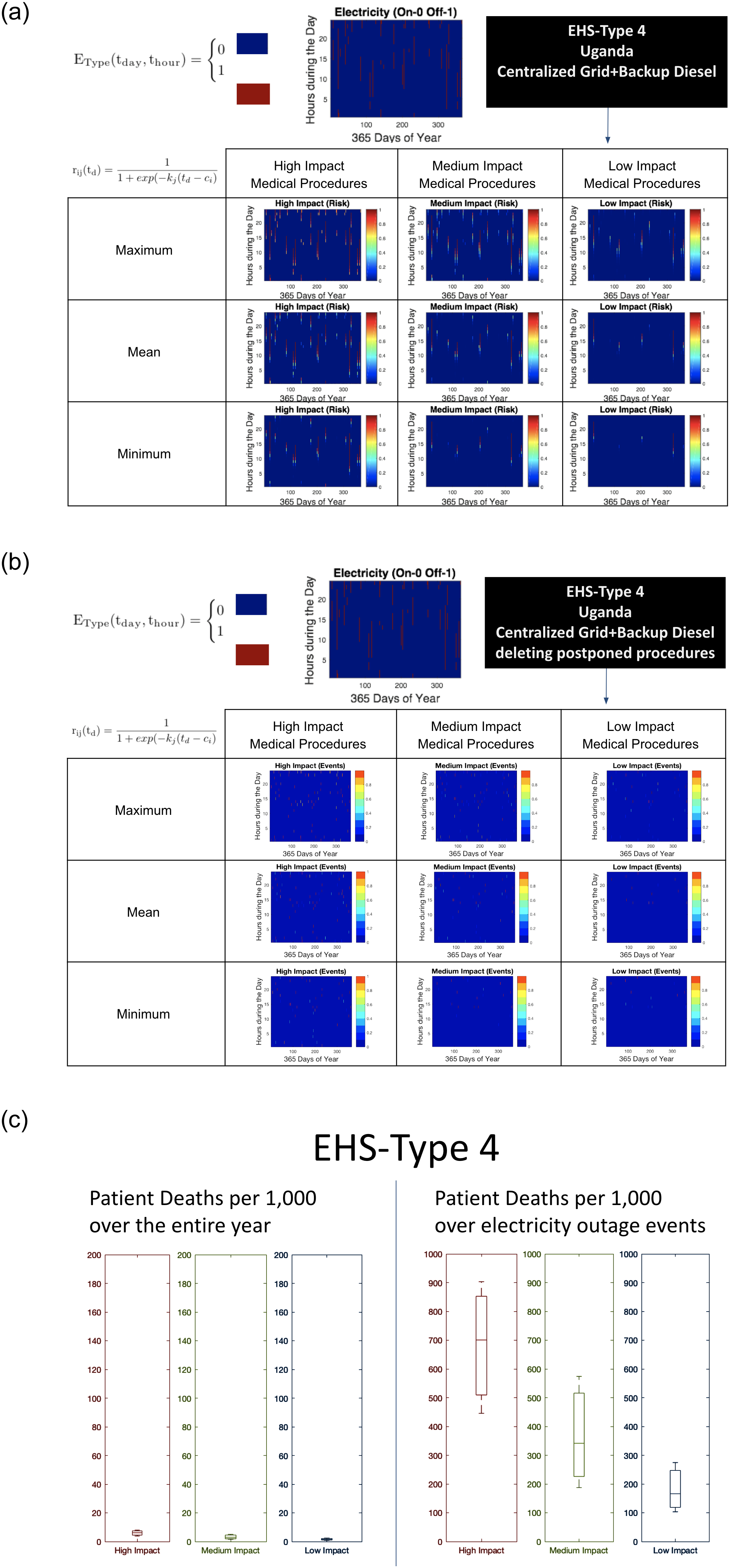

Supplement: S12 Fig — (TIFF) [file pone.0235760.s017.tiff]

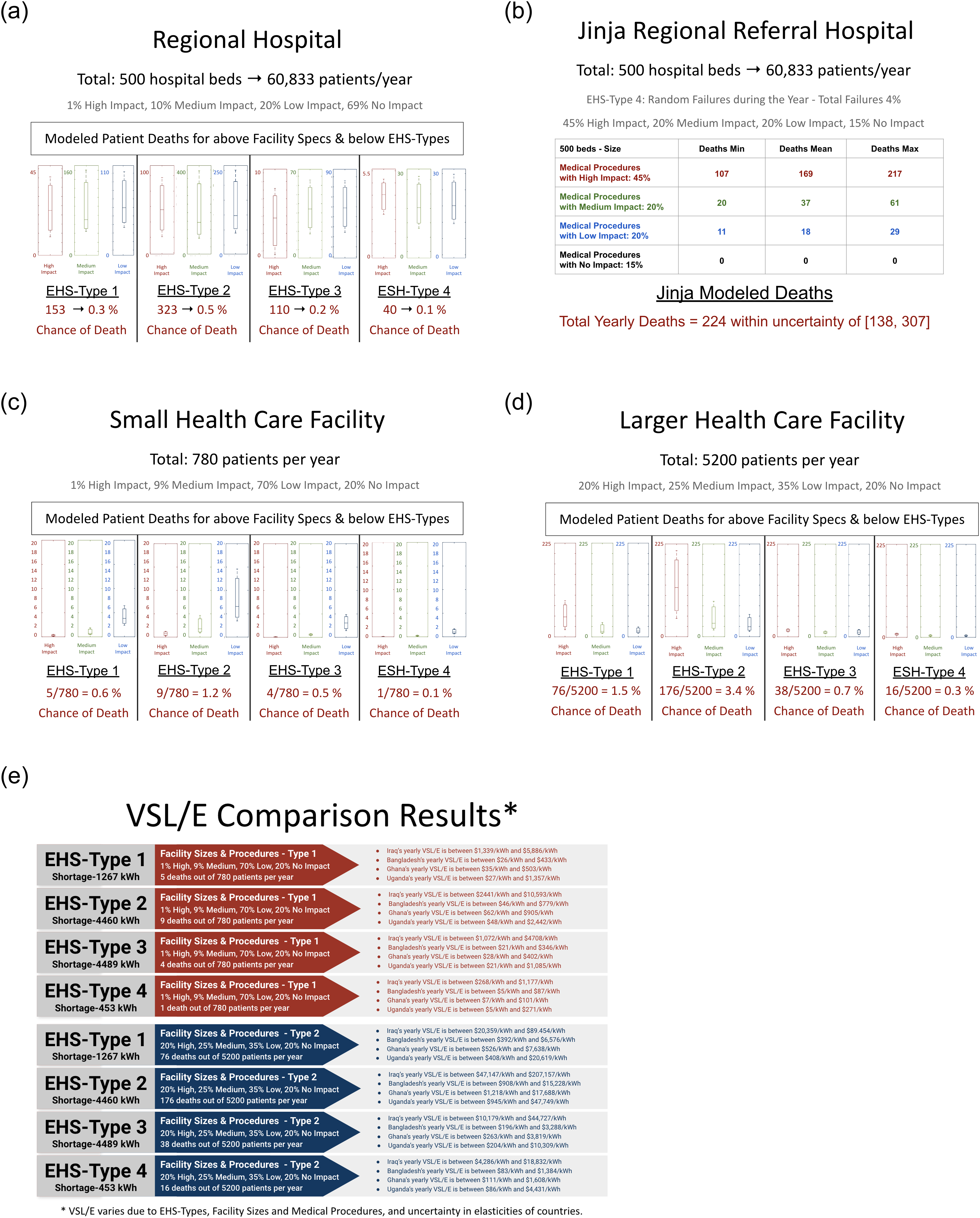

Supplement: S13 Fig — (TIFF) [file pone.0235760.s018.tiff]

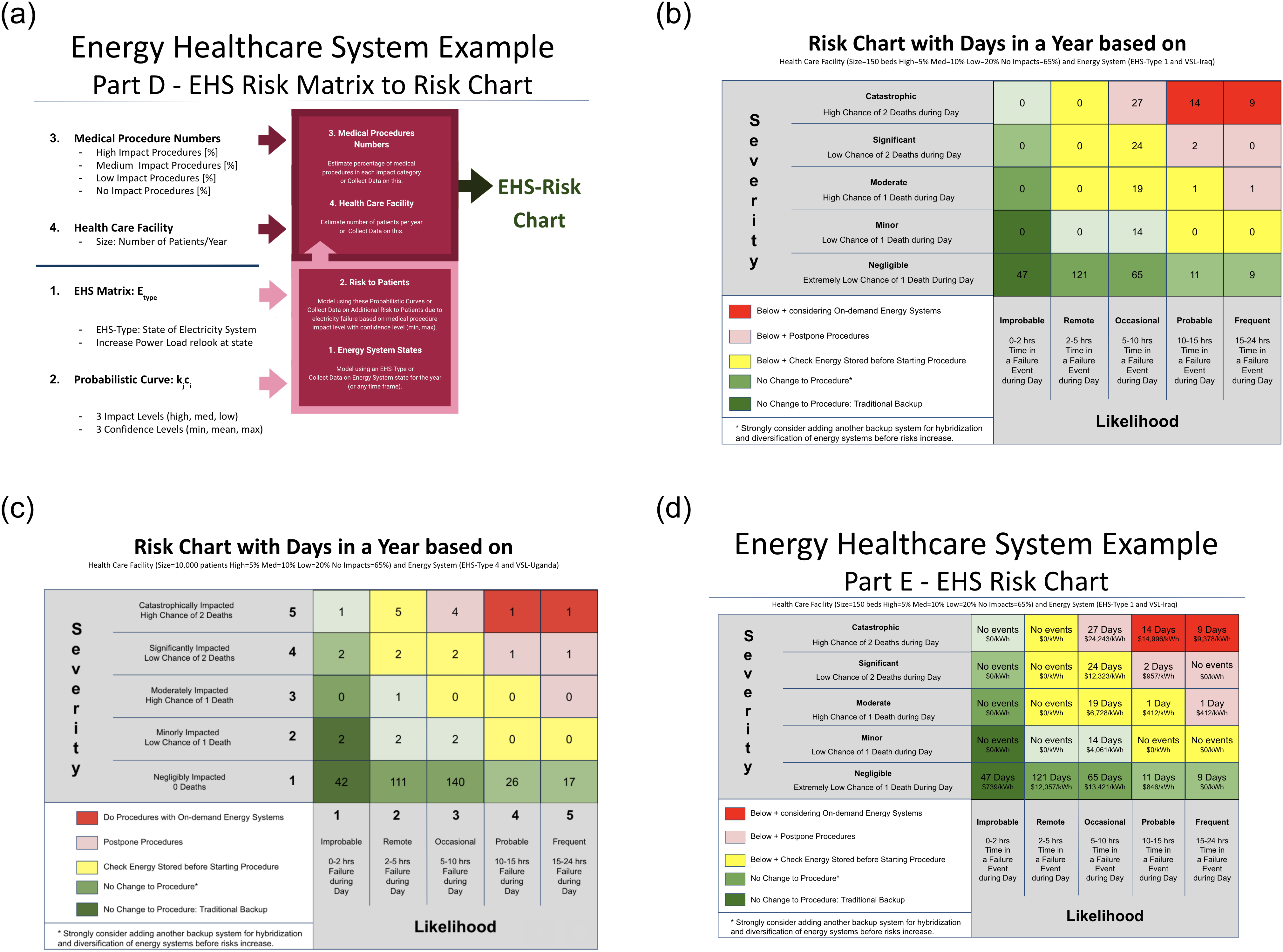

Supplement: S14 Fig — (TIFF) [file pone.0235760.s019.tiff]
