# Supplementary material for: Health care during electricity failure: The hidden costs
Source: PLoS One. 2020 Nov 4;15(11):e0235760. doi: 10.1371/journal.pone.0235760 (PMC7641375; doi:10.1371/journal.pone.0235760)
Supplement: Attachment — Submitted filename: 2019.PLOSONE-Transfer.Rebuttal.Letter.Mechtenberg.pdf [file pone.0235760.s020.pdf]

UNIVERSITY OF
NOTRE DAME
College of Science

Abigail Mechtenberg
208 Jordan Hall of Science, Notre Dame, Indiana 46556
tel **(574) 631-9265** *fax* **(574) 631-8149** *email* **amechten@ND.EDU**

December 31, 2019

PLOS| ONE
c\o Dr. Joerg Heber
email: jheber@plos.org
1160 Battery Street
Koshland Building East, Suite 225
San Francisco, CA 94111 United States

December 31, 2019

Dear Dr. Jo Heber:

We thank the reviewers for their comments on the manuscript and have edited the manuscript to address their concerns.

We believe that the manuscript is now suitable for publication as a transfer from PLOS|Medicine to PLOS|ONE. Please see our table juxtaposing the reviewer comments with our discussion on how it was addressed as our Rebuttal Letter.

Sincerely,

Abigail Mechtenberg
Physics and ND Energy
University of Notre Dame

Reviewer Notes emailed to me from Clare Stone.

**Reviewer #1 Comments**	**Authors' Response**
The article argues for the importance of reducing patient's risks associated with unreliable electricity supply during surgery. The article's position on the issue is to expose all forms or elicitation of all cost variables that will ensure proper utilization of the cost benefit management methodology such that decisions are made based on solutions that reflect reality. The organization of the article follows conventional journal article format. The paper looks promising, however, the following shortcomings are observed:	
Title of article: the full title of article is too long. Shorten the title with deletion of phrase "A value of statistical life lost divided by energy shortage model events into VSL/E risk chart" and make it (phrase) part of the methodology of this work.	Yes, the title is "Health Care during Electricity Failure: The Hidden Costs" However, we potentially typed in the PLOS|ONE text boxes a different long/short title which I am not sure how to change and would like to talk to editors about this.

UNIVERSITY OF NOTRE DAME
College of Science

Abigail Mechtenberg
208 Jordan Hall of Science, Notre Dame, Indiana 46556
tel (574) 631-9265 *fax* (574) 631-8149 *email* amechten@ND.EDU

Abstract-: the abstract needs modification to remove references. An abstract should as much as possible be able to stand alone. Also, the abstract was incomplete because how the primary data were obtained from respective countries was omitted. Update the abstract to ensure it contains essential elements of an abstract listed as follows- purpose, specific objectives, methodology, results and significance/ value of the study.	References removed from Abstract. We have briefly addressed the concern about the missing explanation of how the primary data were obtained from respective countries by adding to the abstract the phrase "through research literature and/or measurements," although this is a complicated methodological issue and many more details are step-by-step explained in Supplemental Materials. The abstract explains the purpose of the paper in terms of providing awareness of health care risks due to electricity failures as well as a model to calculate costs and create recommendations to adapt and mitigate these risks. We ran out of space for more details due to word count. "These electricity failure risks and hidden costs can now be calculated and charted to make medical decisions based on a risk model instead of anecdotal experience." The abstract explains the specific objectives in terms of the problem: "no methodology exists to quantify these risks, the associated hidden costs, nor risk charts to compare alternatives." The abstract explains the methodology as follows: "Two methodologies were created to quantify these hidden costs. In the first methodology, the authors obtained and analyzed a year's worth of hour-by-hour energy failures for four energy healthcare system (EHS) types in four regions (SolarPV in Iraq, Hydroelectric in Ghana, SolarPV+Wind in Bangladesh, and Grid+Diesel in Uganda). In the second methodology, additional patient risks were calculated according to time and duration of electricity failure and medical procedure impact type. Combining these methodologies, the cost from the Value of Statistical Lives lost divided by Energy shortage ($/kWh) is calculated for EHS type and region specifically. The authors define hidden costs due to electricity failure as VSL/E ($/kWh) and compare this to traditional electricity costs (always defined in $/kWh units), including Levelized Cost of Electricity (LCOE also in $/kWh). This is quantified into a fundamentally new energy healthcare system risk chart (EHS-Risk Chart) based on severity of event (probability of deaths) and likelihood of event (probability of electricity failure)." The abstract then discusses the results in terms of the connection between electricity failures and health care using the new VSL/E metric: "VSL/E costs were found to be 10 to 10,000 times traditional electricity costs (electric utility or LCOE based). The single power source EHS types have higher risks than hybridized EHS types (especially as power loads increase over time), but all EHS types have additional risks to patients due to electricity failure (between 3 to 105 deaths per 1,000 patients)." Finally, the abstract discusses the significance of the study in terms of the Conclusion: "These electricity failure risks and hidden healthcare costs can now be calculated and charted to make medical decisions based on a risk chart instead of anecdotal experience. This risk chart connects public health and electricity failure using this adaptable, scalable, and verifiable model."

Abigail Mechtenberg
208 Jordan Hall of Science, Notre Dame, Indiana 46556
tel **(574) 631-9265** *fax* **(574) 631-8149** *email* **amechten@ND.EDU**

Introduction: the introduction needs to explore into background information of the case study countries. What do the countries have in common in terms of health care and energy supply services? The reader needs to be aware of this information on the common characteristics in order to appreciate the results and comparison made in this work	We disagree and hope PLOS|ONE editors agree, but we greatly appreciated the comment. The comment illustrates the confusion that arises when readers focus on the EHS-Type and Region specifics rather than the EHS-Risk Model and Chart's adaptability, scaleability, and verifiability. Consequently, we added these sentences in the Introduction: "There also exists data on electricity outages for various EHS types and countries. [and] This data and other publications focus on the impact of unreliable electricity as it relates to a health care facility or health care services, but typically not on medical procedures." Also, we added Table 1 to explain these four EHS-Types are distinct electricity failure patterns observed that fundamentally build the statistical foundation for key failure patterns which require unique global health care considerations.
Methodology of study -the theoretical concept of energy failure explored in the work is clear. However, there is a leap of assumptions made to arrive at the selection of countries. For clarity, those assumptions need to be stated and explained. Succinct explanation of the assumptions will be helpful for replication of the study by another researcher. Therefore, the methodology requires improvement. Again, how did you obtain the risks due to failure by using qualitative scenarios?	This model is adaptable, scaleable, and verifiable for any location and any EHS-type. In the supplemental materials there are five key parts that explain the steps for adaptability, scaleability, and verifiability: Part A - Electricity Failure Matrix in Figure S6a, Part B - Additional Risk to Patient in Figure S6b, Part C - EHS Risk Matrix Logic, Part D - EHS Risk Matrix to Risk Chart, and EHS Risk Chart. We hope this is clearer in this version. Furthermore, "Once any data is collected, this ESH-Risk Chart methodology and model will adapt to the data (in terms of any resultant energy failure matrices and any probabilistic curves), will scale to the facility type (in terms of percentage of medical procedures placed in impact groups and total number of patients), and will ultimately be verifiable in decision making (calculate the VSL/E costs due to patient lives lost and the energy needed to provide electricity for that medical procedure)" based on the five clear recommendations in the main part of the paper.
Results: section 3.0 was omitted. What is life worth for a patient in Iraq, Ghana, Uganda and Bangladesh, respectively? I think hidden cost is affected by life worth in each country.	The reviewer did not read Supplemental Material Section 0.6 as we discussed that VSL depends on region's economic elasticity and GDP/capita. Hidden costs are more complicated than the reviewer understands. However, we created new charts and graphs in supplemental materials to make these points clearer. VSL/E depends on all three issues as discussed in Supplemental Materials Section 0.7.6 and specifically Figure S13e and as Facility Size grows and percentages of Medical Procedures are more impacted by electricity failures, the VSL/E is much greater than LCOE regardless of EHS-Type and VSL in Region.
Discussion: with scarcity of data on electrical failure and corresponding patient outcome, the VSL/E is affected. How did the authors validate the results?	The authors now clearly argue that this EHS-Risk Chart model is adaptable, scaleable, and verifiable as data becomes available. Furthermore, validity of this research is based on the model's breadth, depth, and scientifically compelling paradigm shift. This theoretical model paradigm is more valid than the existing paradigm of making EHS decisions based on anecdotal experience. The shift away from ignoring EHS-Risk issues due to lack of data should be a call for EHS data collection and mapping cost-benefit decision-making

UNIVERSITY OF NOTRE DAME
College of Science

Abigail Mechtenberg
208 Jordan Hall of Science, Notre Dame, Indiana 46556
tel (574) 631-9265 *fax* (574) 631-8149 *email* amechten@ND.EDU

	possibilities from EHS-Risk Chart. This research increases its validity in the future as it showcases its adaptability, scaleability, and verifiability based on new data collection, including surveys of impact analysis when recommendations are implemented. Paradigm shifts rarely get published due to lack of data, especially in a Data Science driven academic publication world; however, it is imperative and vital that this paradigm shift is published because people are dying unnecessarily. The baby born into a kerosene latern, a story that the World Health Organization uses to argue for electricity, was not due to lack of solar panels, it was due to lack of on-demand lighting because they had a solar panel system, but the batteries were out of charge. Finally, we did explain how the model can be used to evaluate the claim of 150 deaths in six months at Jinja Referral Hospital in Uganda supposedly due to electricity failure alone with more details in Supplemental Materials Section 0.7.5. The claim was found to be possible even though the government and the utility company, UMEME, say that it was not possible. Furthermore, our VSL/E calculations can be used to argue for these five recommendations.
Recommendation and Conclusion: a distinct section for recommendation and conclusion is required as a part of a traditional journal article format. The conclusion should incorporate the key findings from the study or extent to which the objectives were satisfied.	Yes, we added these sections and believe we did this. We look forward to reading and incorporating other reviewer comments as we near in-press publication status. We have so many more recommendations, but we have condensed this to five vital recommendations.
Action required: the author (s) should address all the queries raised in respect of PMEDICINE-D-19-01106 by this reviewer.	Done.

**Review #2's Comment**	**Response to Review #2's Comments**
Although this manuscript by title addresses the very important topic of the impact of electricity failures on health care, after reading the manuscript I did not feel I learned much of substance regarding the topic and how it can be addressed.	I heard the same comment by a close colleague. This prompted us to develop clear and concrete recommendations from this research. Now, we believe that this manuscript is fundamentally presenting the research results with clarity and implications moving forward.
I will provide comments in order through the manuscript and then the supplementary materials, and then make overall comments.	
Abstract:	
I do not believe reference numbers are typically included in an abstract.	Yes, this was changed.
Intro:	
Define abbreviations on first use (e.g. LMIC).	Yes, this was changed.
Electricity failure obviously has devastating effects on surgery. How about the ICU (ventilators)? Emergency dept (x-ray, CT, labs)?	In this article, we are more focused on electricity failure pattern compared to the power level or energy source creating this specific electricity failure. To make this point

UNIVERSITY OF NOTRE DAME
College of Science

Abigail Mechtenberg
208 Jordan Hall of Science, Notre Dame, Indiana 46556
tel (574) 631-9265 *fax* (574) 631-8149 *email* amechten@ND.EDU

	clear, we created more explanations and details. However, the optimal energy system model for creating the EHS-Type used the power load of the health care facility where power load was modeled from the actual medical appliances being used. Recommendation number one deals with this issue exactly, succinctly and hopefully compellingly. Recommendation number two deals with the power load prioritizations in terms of more hybridizations and/or backup electricity systems.
Fig 1: presumably the size of the circle reflects the country population - but this should be stated.	Country population is labeled as size of circle in Figure 1's caption.
"dire straights" (sic): "dire straits" does not seem appropriate for an academic publication.	Deleted and thanks!
Methods:	
Fig 2: it appears there are no power outages at night from ~ day 50 to day 325. Should one thus do surgery at night?	No and we explain this is a result of power load increases during the day.
Results:	
Fig 4: Label the four countries; numerical data below graphs too small to read.	Done.
Fig 5: Five colors in legend but six colors in figure itself.	Done.
Human powered backup - should be in Discussion section.	Done.
Disc:	
First three sentences should be in Intro.	Done.
Define UMEME.	Done.
Last sentence not relevant to topic of this manuscript.	Done.
Supplemental:	
First sentence not relevant.	Done.
Fig S1 & discussion repeats info in body of manuscript	Although this may be true, it is hard to follow the logic and flow of the supplemental materials if readers are going back and forth to understand the context. Maybe if a specific example can be given that would be greatly appreciated.
Fig S4 " " " " " "	Although this may be true, it is hard to follow the logic and flow of the supplemental materials if readers are going back and forth to understand the context. Maybe if a specific example can be given that would be greatly appreciated.
Fig S5 " " " " " "	Although this may be true, it is hard to follow the logic and flow of the supplemental materials if readers are going back and forth to understand the context. Maybe if a specific example can be given that would be greatly appreciated.
Repetitive discussions in the four country scenarios	Although this may be true, if readers in main article skip the other sections and go to a specific EHS-Type, then we thought it would be good to explain the details with the

UNIVERSITY OF NOTRE DAME
College of Science

Abigail Mechtenberg
208 Jordan Hall of Science, Notre Dame, Indiana 46556
tel (574) 631-9265 *fax* (574) 631-8149 *email* amechten@ND.EDU

	assumption that they did not read the earlier EHS-Type summaries
Discussion of VSL is good	Thanks.
Table S6: huge variations in VSL/E depending on elasticity chosen. How to decide which E is most relevant?	They are all relevant. It is important to understand that these uncertainties are huge without a stronger data set which needs to be collected. This is a call for more data collection. It is important for health care providers to understand these recommendations regardless of EHS-Type and Facility Size and Medical Procedure numbers as it relates to EHS-Risk charts and moving forward with recommendations and ultimately new policies as a new policy instrument.
Does the choice of only 1% high impact medical procedures reflect reality in LMICs (e.g. Jinja)? Trauma, acute abdomen, C-sections are likely >> 1% of all procedures in LMICs.	Without more detailed data, we can only validate the parameters in which Jinja Regional Referral Hospital claimed number of deaths. This claim was an electricity failure pattern type of either (1) grid and backup diesel generator (EHS-Type 4), (2) power load shedding for a set of hours each day (EHS-Generic Type referred to in the Supplemental Materials), or (3) a combination of random black outs and scheduled power load shedding. We added the notation in the Supplemental Materials to refer to the Generic Type as power load shedding based on this comment. Furthermore, yes, using EHS-Type 4, assuming Jinja uses the number of hospitals beds as quoted with at least the number of patients per year stated, then 25% high impact would be required to meet this claim. So, the example in the risk chart is one of many EHS-Risk Charts that would be and can be generated.
Analogy comparing medical procedures to a driving trip: this is not appropriate since many medical procedures are not elective; the trip appears to be non-emergent.	Although this is fair critique, we believe the analogy of a road trip for decisions to be made in the furture is imperative. We look forward to further discussion on this point as we can drop this point, but others have found this to be a clear analogy and helpful in understanding our recommendations.
The cost of idling diesel generators is a very important point but more common-sense than dependent on the VSL/E analysis. What may be most cost-effective is a battery backup that kicks in immediately when the electricity fails, allowing the diesel generator to be started (within a minute or two) - thus obviating diesel generator idling time.	No. I am going to have to disagree based on vast experiences, including one that highlights this issue and comes from an event at KNUST Teaching Hospital. It took "a minute or two" for the diesel generator to be started and the patient died while in heart surgery. This argument of "common-sense" is an argument of "anecdotal experience" and should not be acceptable.
Other comments:	
More discussion of human-generated electricity to complete emergency surgery/maintain ICU equipment - perhaps the most immediate solution in some LMIC situations.	We tried, but we do not want to side-track from the main part of the paper. We could add five pages to the Supplemental Materials including CAD designs for locally built devices in Uganda (and why Leslie and Emmanuel were added as co-authors to this version). Please advise. We worry this is already too long.
Need discussion of energy-efficient medical equipment (e.g. CT scan operated by car battery	We tried, but we do not want to side-track from the main part of the paper. We could add five pages to the

Abigail Mechtenberg
208 Jordan Hall of Science, Notre Dame, Indiana 46556
tel (574) 631-9265 *fax* (574) 631-8149 *email* amechten@ND.EDU

NeuroLogica/Samsung) - in addition to suggestions such as disabling all non-emergency electrical outlets during power outages.	Supplemental Materials including wireless examples of how to turn off outlets remotely using an app or at the switch both. Please advise. We worry this is already too long.
Are relevant data actually "suppressed" - or merely not available or not published? If maliciously suppressed, examples should be given.	Yes, we have rewritten this, but we have to be careful in how this is worded. Thank you for this comment.
The three recommendations noted in both the manuscript and the supplemental material are (esp #2 & #3) common-sense - not dependent on the findings of the manuscript.	Respectfully, we know that these recommendations are not common-sense for two reasons. First, if they were common-sense, then they would already exist. Second, they directly emerge from this research collaboration and its results. Therefore, we completely disagree and hope this reviewer does not require us to write out all of our research notes about how we came to these recommendations from doing this research step-by-step. Again if they are so common-sense, then why are they not being done?
Overall - needs close proofreading.	Done
Make text more readable (shorter/clearer phrases & sentences)	Done
Correct typos	Done as much as possible. The paper was edited more than 3x following, and according to reviewer comments.
Less use of "word" in parentheses	Done
Data = a plural noun	Done.
Figures have legends but additional explanation in text	Done.
Is death the only measurable outcome?	For now, yes. But we are looking into DAYS.
Keep order of the four countries consistent	We are trying to distant the readers from the countries and energy sources and to get the readers to think about the statistics and electricity failure patterns. This is broader and deeper as it is a paradigm shift away from "common-sense" arguments which look like anecdotal experience-based arguments and move towards scientifically-based data collection to adapt, scale, and verify this EHS-Risk Model.

Review #3's Comment	Response to Review #3's Comments
This is an interesting study on electricity failures on health care in terms of hidden costs and risks. However, there are a few major issues needing attention.	
1) Firstly, the study is mostly a theoretical/simulation exercise without the support of real world data such as patient additional risk due to failure, therefore it is a proposed risk model or framework without any validation or proof.	We show clearly how to validate Jinja's claim (and any other claim made that the government tries to suppress - and yes, this is where suppression of data comes and we have to make this comment private and not public). No proof can be given until data is collected. This is more than a

UNIVERSITY OF NOTRE DAME
College of Science

Abigail Mechtenberg
208 Jordan Hall of Science, Notre Dame, Indiana 46556
tel (574) 631-9265 *fax* (574) 631-8149 *email* amechten@ND.EDU

	theoretical/simulation exercise, this is broader and deeper paradigm shift away from "common-sense" arguments which look like anecdotal experience-based arguments and move towards scientifically-based data collection to adapt, scale, and verify this EHS-Risk Model. Anyone who understands risk, electricity failures, and patient survival models, can understand how broad and deep this EHS-Risk Model is as a paradigm shift.
2) The cost-benefit analysis is too simplistic and could be more comprehensive to involve more potential factors in the analysis. For example, most hospitals have emergency secondary backup power supply to their surgical rooms. Have authors considered this in their risk models? This is only one potential factor and could be many more.	Yes, again they do not understand the fundamental electricity failure patterns for various energy systems. We have considered over a hundred: both simulations, qualitative interviews, and measurements of various energy systems (including merry-go-round generators for children's wards and why we have the hybridization recommendation). The energy system is not the issue. The electricity failure pattern is the issue.
3) The manuscript looks like a technical report and far from a paper.	That is not true, but it depends on what allows for a research paper. This is a call for a fundamental paradigm shift which is a paper. A technical paper is one that tinkers at already existing scientific knowledge and progresses some little bit of knowledge for a variable or two. This is not a technical paper. In fact, this bridges two fields together that have never been brought together (electricity failure patterns and health care), with a new metric VSL/E that will change the way people think about costs of electricity. Furthermore, if in the future, there are more electricity failures due to cyberattacks or climate change, then this will become a way to justify paying people to bring food waste for electricity or even biking, within the limits of such.
4) The paper is a bit technical overall and simplistic in cost-benefit analysis without any validation therefore is not suitable for publication in PLOS Medicine.	Yes, and it was transferred to PLOS-ONE.

**Review #4's Comment**	**Response to Review #4's Comments**
This paper discussed the implications of electricity failure on healthcare service delivery using some case studies from some developing countries. The idea behind the paper is quite interesting given the deplorable state of power in many developing countries. The study was well motivated, although the knowledge gaps were not clearly highlighted. The major concern I have for this study is the approach.	
The authors would have done a better job if the following were taken cognizance of:	
* Healthcare facilities are not homogenous. Some are dispensaries,while others are tertiary medical centers. The impacts of electricity failure would differ based on the category that these healthcare facilities belong. What applies in a private medical center would be different from a public medical center.	This is fundamentally about electricity failure patterns and outcomes from medical procedures that are known by the private medical center and public medical center. The EHS-Risk Chart is generated from (1) electricity failure pattern, (2) health care size and experience of patient outcomes when electricity fails from on-site data collection which probably cannot be shared, but known internally, and (3) country VSL. It can be adjusted in time

Abigail Mechtenberg
208 Jordan Hall of Science, Notre Dame, Indiana 46556
tel **(574) 631-9265** *fax* **(574) 631-8149** *email* **amechten@ND.EDU**

	step moving forward. It can be adjusted in patient risk function (c,k and type) moving forward.
** There would be a need to focus on certain region of a country and carry out some evaluation of the impacts of electricity outages on efficiency of healthcare service delivery. It would make more meaning if medical records were accessed in order to determine how many died due to power outage or what strategies are in place to savage the deadly implications of power outage. Instead of addressing this problem from a macro level, a micro analysis would make more meanings with essential policy implications.	This has been tried, but without a large call for data collection, this is impossible. If one considers what happened at Jinja Referral Regional Hospital, one would know that the government did not want the public to know this data. Consider what is happening in California due to electric utility turning off the electricity and the patient dying due to electricity failure at home? Unlike the US, the political powers facing an uprising of people upset cannot afford this data leaving the local hospital. However, a larger study could be done such that individual hospitals and health care facilities identities were hidden. This paper could help this call for R&D funding.
***. The underline assumptions of electricity generation and risks associated with power outage are too simplistic given that there are different heterogeneity within these countries in terms of the prevailing disease, medical costs and expert knowledge possessed by the medical staff.	Again see above.
